# Advances in nanomechanical property mapping by atomic force microscopy

**DOI:** 10.1039/d5na00702j

**Published:** 2025-08-26

**Authors:** Ricardo Garcia, Jaime R. Tejedor

**Affiliations:** a Instituto de Ciencia de Materiales de Madrid, CSIC c/ Sor Juana Inés de la Cruz 3 28049 Madrid Spain r.garcia@csic.es

## Abstract

AFM-based mechanical property measurements are widely used in energy storage, polymer science, mechanobiology or nanomedicine. Mechanical properties are determined by expressing the experimental force in terms of a contact mechanics model. A nanomechanical map is generated by representing one or more mechanical parameters as a function of the tip's spatial coordinates. Force spectroscopy modes might be separated into two categories, adhesion and indentation. Here we describe the principles of AFM-based indentation modes to generate spatially resolved maps of the mechanical properties at the nanoscale. The review provides an update on the progress in nanomechanical mapping since 2019. The focus is on quantitative accuracy, spatial resolution, high-speed data acquisition, machine learning and viscoelastic property mapping. Two advanced applications which emerged from AFM-based indentation modes, nanomechanical tomography and volume imaging of solid–liquid interfaces, are also described.

## Abstract 2

AFM-based mechanical property measurements are widely used in energy storage, polymer science, mechanobiology or nanomedicine.

## Graphical abstract

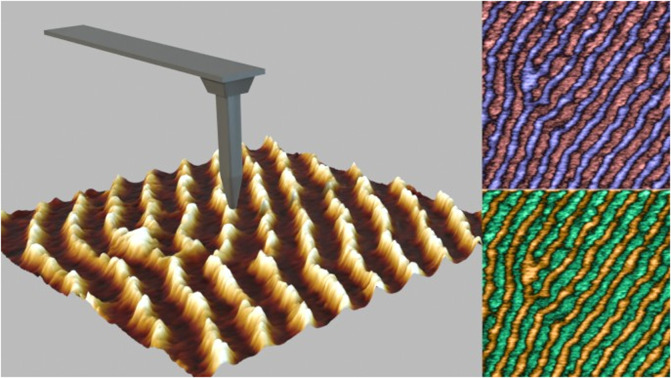

## 1. Introduction

The atomic force microscope (AFM) has become the dominant technique to characterize mechanical properties at the nanoscale. The AFM might be considered a mechanical microscope which transforms the interaction force between the tip and the sample surface into an out-of-plane deflection of the cantilever-tip transducer.^1,2^ In a further processing step, the instantaneous deflection is transformed back into an instantaneous value of the force. The interaction force might be expressed in terms of parameters associated with the mechanical properties of the sample. Therefore, the AFM is ideally suited to measure mechanical properties of surfaces, interfaces and nanomaterials at the nanoscale.^3–15^ The generation of spatially-resolved mechanical property maps at the nanoscale is called nanomechanical mapping.^11^ It has been extensively improved since the first pioneering measurements performed more than 30 years ago.^3,4^

**Fig. 1:**
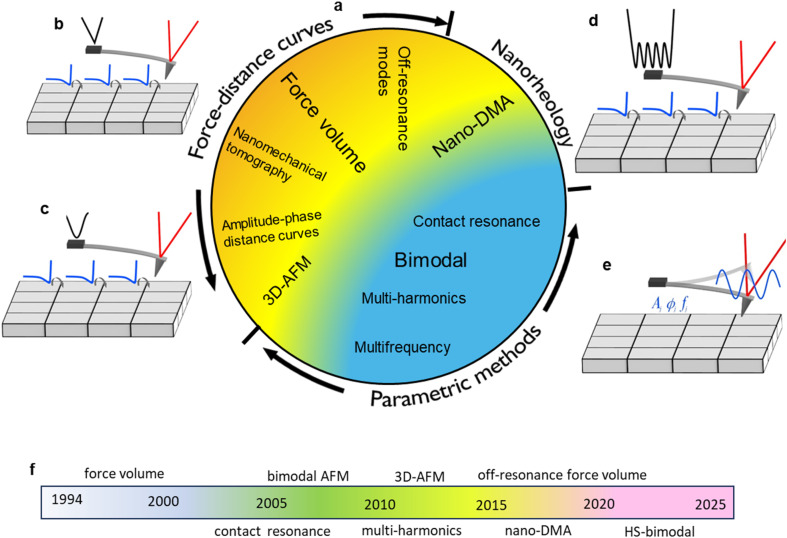
Nanomechanical mapping. (a) Classification of popular and emerging AFM-based indentation methods. A spatially-resolved map of mechanical properties is generated by performing measurements in each pixel of the sample surface. (b) Scheme of force volume (triangular *z*-displacement). (c) Scheme of force volume off-resonance (sinusoidal *z*-displacement). (d) Nanorheology. (e) Scheme of nanomechanical mapping by using a parametric method. *A*, *ϕ* and *f* are, respectively, the amplitude, phase shift and resonant frequency of the tip. (f) Milestones in the evolution of nanomechanical mapping (indentation modes). HS stands for high-speed. The time scale represent approximative dates.

Several AFM modes have been developed to measure mechanical properties.^3–20^ These modes might be broadly divided into two major categories, indentation^3–13,15–18^ and adhesion.^14,19^ AFM-based single-molecule force spectroscopy,^14^ ringing mode^18^ or van der Waals interaction mapping^21,22^ belong to the latter category. Indentation and adhesion property data might be combined to generate multiparametric images of heterogenous materials at the nanoscale.^14,23^ Indentation and adhesion modes differ in sample and tip preparation protocols, observables and theoretical backgrounds. For these reasons, it is convenient to discuss them separately. This review is focused on modes based on applying a deformation to the sample surface (indentation). Fig. 1 illustrates the variety of nanomechanical property mapping modes based on producing a reversible deformation in a material.

Mechanical properties are obtained by analysing the repulsive component of the interaction force or by measuring its effect in the cantilever dynamics. It has to be emphasized that sample deformation might not involve an irreversible modification of the sample. In fact, a nanomechanical map should be obtained under operating conditions that avoid any permanent damage of the sample and the tip. Nanomechanical mapping proceeds sequentially. First, the mechanical property is measured on a single point of the surface, then a map is completed by repeating the process in the other points of the surface. As a consequence, single and sequential mechanical property measurements are performed with the same contact mechanics model.

This review introduces the advances in nanomechanical property mapping and its applications since 2019. It complements and updates a previous review on nanomechanical mapping.^11^ The present review is divided into four sections. The first section provides a classification of nanomechanical mapping modes in three groups, force–distance curve, parametric and nanorheology. The second section presents the theoretical concepts needed to understand the relationship between the AFM observables and the mechanical properties of a material. The third section addresses issues related to macro and nanoscale measurements, quantitative accuracy, spatial resolution, and throughput. The last section illustrates several applications in materials science, energy storage and cell biology. Notably, it introduces two advanced modes for generating three-dimensional images of materials and interfaces. Finally, we conclude with some thoughts about the technological challenges that might shape the evolution of nanomechanical mapping.

## 2. Nanomechanical mapping modes

Fig. 1a shows a classification of AFM-based indentation modes. The modes are subdivided in three groups, force–distance curves, nanoscale rheology and parametric methods. Fig. 1b and c illustrates the concept of nanomechanical mapping by using force–distance curves. Fig. 1d and e illustrates the methods based on studying the effects of the sample's mechanical properties on the tip's oscillation parameters. Fig. 1f shows some milestones in the development of nanomechanical mapping modes.

### 2.1 Force volume

Force volume is a nanomechanical mapping mode based on acquiring a force–distance curve (FDC) in each pixel of the sample surface (Fig. 1b and c). Those curves might be transformed into maps of mechanical parameters by fitting the curves to a contact mechanics model. Force–distance curves are generated by modulating the tip-sample distance and recording the cantilever's deflection as a function of the distance. Force–distance curves are usually recorded in both directions, that is, by approaching and retracting the tip from the sample surface. The approach and retraction components of a FDC might contain complementary information on the sample mechanical properties.

**Fig. 2:**
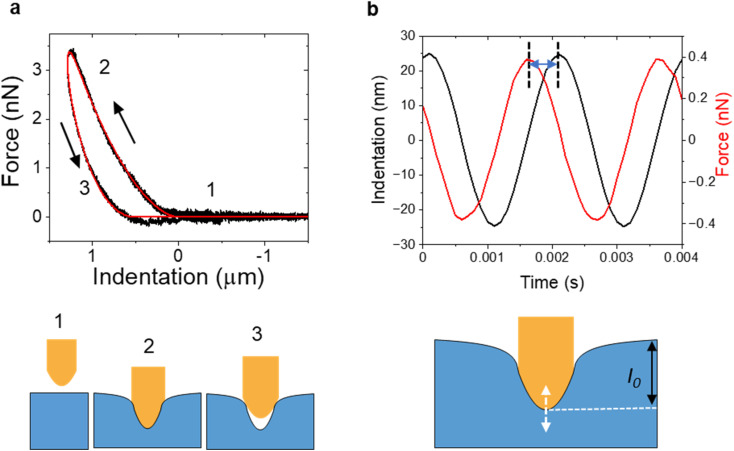
(a) Force–distance curve with approaching and retraction sections obtained on a live HeLa cell (top panel). Experimental data in black and a theoretical fitting in red. The arrows indicate the direction of the tip displacement. Bottom panel, schemes of the tip-sample distance for the positions marked in the FDC. (b) Force and *z*-displacement in a nanorheology experiment. The time lag between *z*-displacement and force is marked. Bottom panel. A schematic diagram of the experiment. The tip is oscillated with respect to the indentation *I*_0_ defined by the set point force value.

Fig. 2a shows an example of a FDC obtained in a live mammalian cell. The FDC shows that approach and retraction sections of the curve do not overlap. A hysteresis cycle is a direct indication of the presence of inelastic and energy dissipation processes in the material.^2^ In this case, the hysteresis indicates the viscoelasticity of the cell. A viscoelastic response generates a delay in the sample deformation with respect to the tip's retraction (Fig. 2a, bottom panel (3)).

The modulation of the tip-sample distance (*z*-distance) might be achieved by mechanical, magnetic or photothermal methods. Mechanical methods operate by modulating the *z*-distance with triangular or sinusoidal waveforms. The first nanomechanical property maps were acquired by modulating the *z*-distance with a triangular signal.^3,4^ This type of modulation provided a constant tip's velocity which facilitated data interpretation. Those maps were called force volume.^4^ Despite of its popularity, the use of triangular waveforms had some limitations in the imaging rate.^11^ A triangular waveform contained a large number of higher harmonics. Those harmonics could be coupled to some of the mechanical resonances of the *xyz* piezos. In addition, the discontinuity of the tip's velocity at the turning point might cause an artefact in the cantilever deflection. That artefact became more pronounced at high velocities. Those issues motivated the introduction of sinusoidal signals.^16,17^ Sinusoidal modulations have improved the imaging rate and opened the development of several off-resonance excitations.^24–26^

The term off-resonance indicates that the frequency of the signal that modulates the tip-sample distance is significantly lower than the frequency of the first flexural frequency of the cantilever. In particular, by using a photothermal force to drive the cantilever-tip's *z*-displacement,^27–29^ it was possible to reach a rate of 0.4 frame-*per*-second (512 × 256 pixels) to imaging surface topography features.^24^

The term force volume mode was applied to describe the first nanomechanical maps obtained by using triangular waveforms.^4^ The introduction of sinusoidal waveforms led to a variety of names. However, we favour a physical-based definition.^11^ In this way, all the modes based on the acquisition of a FDC on each point of the surface belong to the force volume category irrespective of the type of waveform and/or cantilever actuation.

Force–distance curves might be also obtained by operating the AFM with amplitude modulation feedback (tapping mode).^2,30^ In this case, the tip is driven at the first flexural resonance while the tip-sample distance is modulated with a triangular waveform at frequency several orders of magnitude lower than the resonant frequency. A force–distance curve is obtained by measuring simultaneously the changes on the amplitude and phase-shift of the tip's oscillation at each distance.^2,31,32^ Three-dimensional AFM (3D-AFM)^33^ makes an extensive application of FDCs obtained in dynamic AFM modes^34^ to characterize solid–liquid interfaces at the molecular scale.^33,35–43^

### 2.2 Nano-DMA

In AFM-based nanorheology, the tip is first approached towards the sample to reach a predefined set point force value (1–20 nN), then an oscillatory signal is applied to either the cantilever or the *z*-piezo while the tip is in contact with the sample^44–49^ (Fig. 1d). The tip oscillates with respect to an indentation *I*_0_ (100–500 nm) defined by the set point force value (Fig. 2b).

The resulting low-amplitude oscillating motion of the tip (10–50 nm) is recorded and transformed into a force as a function of time. The viscoelastic properties of the material are encoded in the time lag between the tip's indentation and the applied force (Fig. 2b, see also Section 3.2). The frequency of the oscillatory signal might be changed from a few to hundreds of Hz. Those modes were inspired by macroscopic rheology measurements, such as dynamic mechanical analysis (DMA),^50^ as a consequence, those modes are also called nano-DMA.

### 2.3 Parametric modes

Alternatively, nanomechanical properties and maps might be obtained by driving the cantilever-tip system at its resonant frequency without acquiring a FDC. The observables of the tip's oscillation such as the amplitude, the phase shift or the frequency shifts are recorded on each point of the surface (Fig. 1e). Bimodal AFM,^5,18,51^ contact resonance AFM^12,13,52–54^ or multi-harmonic AFM approaches^20,55–57^ belong to this category. Those methods are called parametric because the mechanical properties are parameterized in terms of the tip's oscillation parameters.

In bimodal AFM, compact analytical expressions enable to transform the observables of the two excited eigenmodes of the microcantilever into mechanical properties.^51,58–60^ This feature makes bimodal AFM compatible with high-speed imaging.^60^ The bimodal observables are the amplitude and phase shift of the first mode and the amplitude and phase or frequency shift of the second mode (see below).

Contact resonance AFM and multi-harmonic AFM require the use of numerical methods to relate the observables to the mechanical parameters. In contact resonance AFM, the observables are the resonant frequency of the tip in contact with the sample and the quality factor of the resonance curve.^12,13^ In multi-harmonic AFM the observables are the mean deflection, the amplitude and phase shift and the indentation. A nonlinear least squares fitting is needed to relate those observables to the nanomechanical properties.^13,20^

Parametric modes should not be confused with multiparametric imaging. The latter term describes the generation of maps of several mechanical parameters. Those parameters might include the modulus, the adhesion force or single-molecule recognition properties. The maps are generated by processing FDCs obtained from force volume.^14,23^

AFM phase imaging is very useful to generate high-spatial resolution compositional contrast maps of heterogenous surfaces.^11,61–66^ Those maps are understood in terms of energy dissipation process.^11,67,68^ However, those maps do not provide quantitative information on constitutive material properties. For that reason, AFM phase imaging has not been included in the classification given in Fig. 1.

### 2.4 Nanomechanical mapping modes and brand names

The majority of nanomechanical mapping measurements are performed with commercial instruments. Each commercial AFM model has its unique hardware and software features. This has led to a variety of brand names. Commonly, AFM users refer to a force spectroscopy measurement in terms of the brand name instead of referring to the physical process. This fact generates some confusion and makes it difficult to compare data obtained from different commercial systems. Table 1 summarizes some brand names and the relation to a nanomechanical mode.

**Table 1:** Nanomechanical mode and brand names

Brand name	Nanomechanical mode
AM-FM	Bimodal AFM with an AM feedback (1st mode) and a FM feedback (2nd mode)
Fast force volume	Force volume. A FDC is obtained with a sinusoidal waveform generated by piezo actuation
Peak force QNM	Force volume. A FDC is obtained with a sinusoidal waveform generated by piezo actuation
Pin point nanomechanical mode	Force volume. A FDC is obtained with a triangular waveform generated by piezo actuation
Quantitative imaging	Force volume. A FDC is obtained with a triangular waveform generated by piezo actuation
WaveMode	Force volume. A FDC is obtained with a sinusoidal waveform generated by photothermal actuation

## 3. Interaction forces and mechanical parameters

Table 2 shows the most common quantities measured in nanomechanical mapping. Among them are the Young's modulus, the viscosity coefficient and the storage and loss moduli, other quantitates such as the adhesion force or the energy dissipated might also be plotted in a nanomechanical map. The relevance of the latter quantities to provide compositional contrast in heterogenous surfaces and to understand interfacial processes should not be underestimated.^11,14,63–70^ Adhesion force and the energy dissipation measurements depend on the tip's size and geometry. For that reason, they have not been included in the table.

**Table 2:** Mechanical parameters

Parameter	Symbol	Definition
Young's or elastic modulus	*E*	Proportional factor between the stress (force per unit of area) and the strain (change of length per unit of length) in a uniaxial deformation
Shear or torsional modulus	*G*	Proportional factor between the shear stress (force per unit of area parallel to the surface) and shear strain
Poisson's ratio	ν	Ratio between lateral and longitudinal deformations
Relationship between Young's and shear moduli	*E* = 2 *G* (1 + *ν*)	
Interfacial stiffness		Elastic quantity. Slope of a force–distance curve. It is a pseudo material property because it depends on the geometry
Complex modulus	E* = *E*′ + *iE*′′	Viscoelastic quantity
Storage modulus	*E*′	Viscoelastic quantity. Real component of the complex modulus. It is proportional to the average energy stored per unit of volume of the material during a cycle of deformation
Loss modulus	*E*′′	Viscoelastic quantity. Imaginary component of the complex modulus. It is proportional to the energy dissipated per unit of volume of the material during a cycle of deformation
Loss tangent	tan *φ*	Ratio between the loss and storage moduli. It is independent on the contact area
Viscosity coefficient	*η* or *η*_G_	Proportional factor between the shear stress and the velocity
Relationship between viscosity coefficients	*η*_e_ = 3*η*	Relationship between shear and elongational viscosity coefficients for an isotropic material
Retardation time	*η*/*E*	Ratio between the viscosity coefficient and the elastic modulus. It applies to Kelvin–Voigt materials
Scaling modulus	*E*_0_	Viscoelastic parameter. In the limit *γ* → 0, *E*_0_ coincides with the Young's modulus. In the limit *γ* → 1, *E*_0_ is proportional the viscosity coefficient
Fluidity coefficient	*γ*	Viscoelastic parameter. It varies between 0 (solid) and 1 (liquid)
Relationship power-law rheology and viscosity	*η*_e_ = *E*_0_*t*_0_	

It should be emphasized that, irrespective of the AFM mode, the calibration of the force constant of the cantilever is essential to provide accurate mechanical property values. Currently there are several contactless methods that facilitate the calibration of fundamental^71^ and higher eigenmodes force constants.^72^

**Fig. 3:**
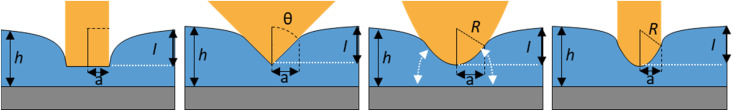
Schematic diagrams of the most common axisymmetric tip geometries in AFM nanomechanical mapping. From left to right, cylinder, conical tip, paraboloid and nanowire. The sample thickness *h*, tip geometry parameters, indentation *I* and projected radius of the contact area *a* are illustrated. The bottom stiffness effect is schematized for a paraboloid (white arrows).

Material property values are determined from either the dependence of the interaction force with the distance (time) or from the effect of the force on the tip's oscillation parameters. A rigorous description of the relationship between interaction forces and/or tip's oscillation observables in terms of mechanical property parameters requires an introduction to contact mechanics models and to the theory of nanomechanical mapping. In this review, the relationship between mechanical properties, interaction forces and observables will be illustrated for a few representative cases. All the expressions were deduced by assuming an axisymmetric tip. Fig. 3 illustrates four common axisymmetric tips, conical, paraboloid, cylinder and nanowire (cylindrical body capped by a half-sphere).

### 3.1 Elastic materials

For elastic materials, the force *F*_ts_ exerted by a tip on a material as a function of the indentation *I* is given by^11,73^

1 *F*_ts_(*I*) = *αEI*^*β*^

where *E* is the Young's modulus of the material, *α* is a coefficient that depends on the tip's geometry and the Poisson's ratio (*ν*), and *β* is a coefficient that depends on the tip's geometry (see coefficients in Table 3). The above expression assumes that the elastic modulus of the material is much smaller than the one of tip. As an example, for a conical tip of semi-angle *θ*, the force is given by2
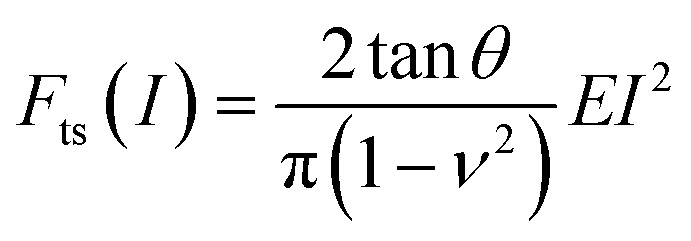


**Table 3:** Coefficients to calculate the force applied by an axisymmetric tip

Tip's geometry	*α*	*β*	*a* contact radius (semi-infinite material)
Cylinder of radius *R*	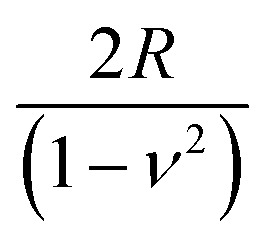	1	*R*
Cone (half-angle *θ*)	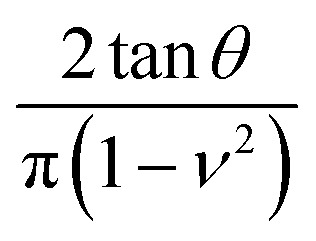	2	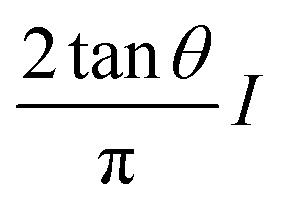
Paraboloid of radius *R*	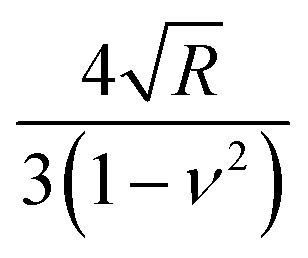	1.5	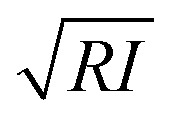

The force exerted by a nanowire of radius *R* is obtained by combining the force of a paraboloid (parab) with the force of a cylinder (cyl),

3a *F*_ts_(*I*) = *F*^parab^_ts_ (*I*) *I* ≤ *R*

3b *F*_ts_(*I*) = *F*^parab^_ts_ (*R*) + *F*^cyl^_ts_ (*I* − *R*) *I* > *R*

In force volume, the elastic modulus might be determined by fitting the experimental force–distance curve with an equation like eqn (1).

In bimodal AFM (configuration AM-open loop), the Young's modulus is obtained by using an analytical expression that links the bimodal observables with the modulus^63^4
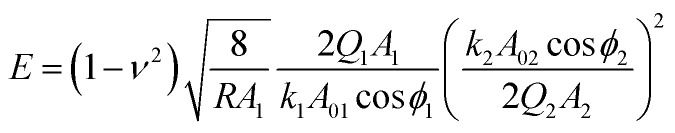
where *A*_*i*_, *A*_0*i*_, *Q*_*i*_ and *ϕ*_*i*_ are respectively the amplitude (set-point and free), quality factor and phase shift of eigenmode *i*. Those observables are readily provided the instrument.

### 3.2 Viscoelastic materials

Several models were developed to describe the viscoelastic properties of materials.^74,75^ In general, viscoelasticity complicates the deduction of analytical expressions of the force in terms of mechanical parameters. For that reason, we provide the force equations for Kelvin–Voigt and power-law rheology models.

The Kelvin–Voigt model is one of simplest linear viscoelastic models. It is represented by a spring in parallel with a dashpot.^74^ Power-law rheology has emerged as the most accurate model to describe the mechanical response of mammalian cells.^76–79^

We note that the force expressions found in general references on viscoelasticity are not the ones that should be used in AFM. To obtain the force exerted by the tip, the relaxation function (viscoelastic model) must be integrated with a function that expresses the change of the contact area with the indentation.^11^ The integration gives the so-called three-dimensional Kelvin–Voigt^73,80^ and power-law rheology force models.^78^ The force exerted by an axisymmetric tip which indents a Kelvin–Voigt material at a constant velocity (*v* = *I/t*) is given by^73^5
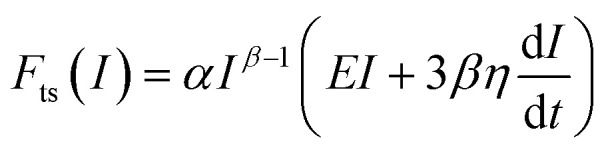
where *η* is the viscosity coefficient associated with a shear force. This is the most common viscosity coefficient, however, in an AFM-based indentation measurement, the force is applied perpendicular to the material surface (stretch or compression). The viscosity coefficient associated with those forces is called elongational or Trouton's coefficient of viscosity *η*_e_*.*^74^ (see Table 2). The force exerted at a constant velocity by a conical tip on a power-law material is given by^78^6
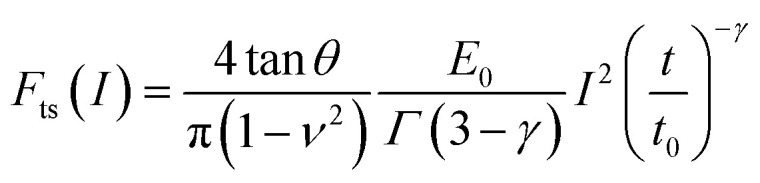
where *Γ* is the Euler gamma function; *E*_0_ is a scaling factor which has been identified as the elastic modulus of the material at time *t*_0_ (commonly *t*_0_ = 1 s). A value of the fluidity coefficient *γ* = 0 defines an elastic solid of Young's modulus *E*_0_ while a value *γ* = 1 indicates a Newtonian viscous liquid. The relationship between the viscosity coefficient and *E*_0_ is given in Table 3.

We note that eqn (5) and (6) are only valid for the approaching section of the FDC. Full expressions of the force for Kelvin–Voigt and power law rheology models are found in.^49,78^ Viscoelastic parameters are determined by fitting an experimental force–distance curve to the theoretical expressions.

In AFM nanorheology, the storage and loss moduli are given by^44^7
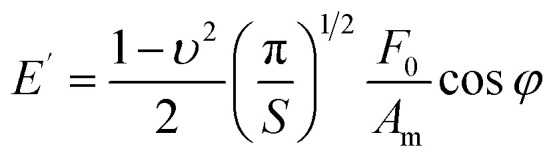
8
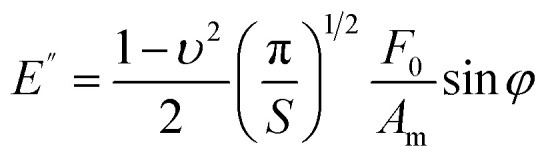


*F*_0_ and *A*_m_ are the amplitudes, respectively, of the force and tip's displacement; *S* (=*πa*^2^) is the projected contact area and *φ* is the phase shift between the deflexion (force) and the *z*-displacement (Fig. 2b). In AFM, the force is commonly applied perpendicular or near to the sample surface. For that reason, the Young's nodulus is most common. Table 2 gives the relationship between compressive and shear modulus.

A useful quantity to characterize viscoelasticity is the loss tangent (tan *φ*) which is defined as the ratio between the energy dissipated *E*_dis_ with respect to the energy stored in the sample (per period).^74^ For an oscillating tip, the loss tangent is the ratio between loss and storage moduli9
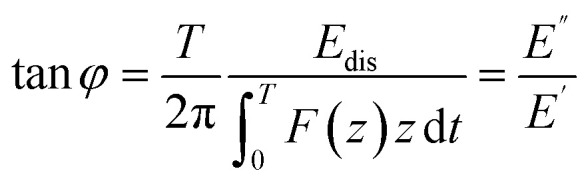
where *T* is the period of the tip's oscillation. The loss tangent is independent of the contact area. For that reason, it is a very useful parameter to compare AFM and macroscale methods.^81^ For a Kelvin–Voigt material the loss tangent might also be obtained by^60,82^10
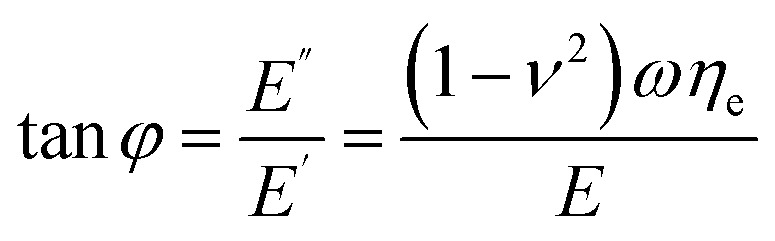
In bimodal AFM^60^ the loss tangent is given by11
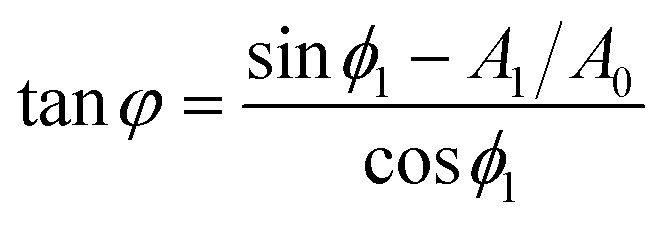


The above expressions were deduced under several assumptions.^2,11,83^ Specifically, (1) the deformation of the sample should be small with respect to the radius of the projected contact area; (2) the force applied by the tip should be perpendicular to the surface, that is, shear forces are not considered; (3) the sample is semi-infinite and (4) the Young's modulus of the tip is significantly larger than the one of the sample. In addition, the measurements should be independent of the tip's mechanical and geometrical properties. Any cross-talk between the topography and the mechanical properties of the sample should be avoided.

Table 3 shows the coefficients needed to calculate the force as a function of the indentation and the contact radius for the most common AFM tip's geometries such as conical, paraboloid and flat cylinder (Fig. 3). The contact radius is referred with respect to the projected contact area. The contact radius has also correction terms associated with the finite thickness of the sample.^11^ Those corrections were not included in the table because they are, in general, negligible.

## 4. Bottom stiffness effect

In many AFM experiments, the sample of interest is deposited, attached or adsorbed on a rigid support (substrate) such as mica, silicon or glass. The finite thickness of the sample gives rise to the bottom stiffness effect. This effect describes how the force applied by the tip propagates through the material to reach the substrate and bounces back towards the tip. The net result would be an increase of the force applied/measured by the tip.

This effect might affect the measurements performed in materials such as nanoparticles, biomolecules, thin polymer films or cells. As a consequence, the use of semi-infinite contact mechanics model such as Sneddon^84^ or Hertz to fit the AFM data obtained on those materials might overestimate the value of the modulus. Bottom-effect analytic correction models were developed to determine the true values of mechanical parameters of a finite-thickness layer deposited and/or attached to a rigid support.^78,80,85–87^ Those models were validated by finite element model simulations,^86–89^ additional theoretical contributions^90,91^ and AFM experiments performed on self-assembled monolayers,^92^ lipid bilayers^93^ and cells.^94,95^

### 4.1 Bottom-effect correction model

The expression of the force exerted by an axisymmetric tip on a finite-thickness elastic material can be obtained by re-writing eqn (1) as12
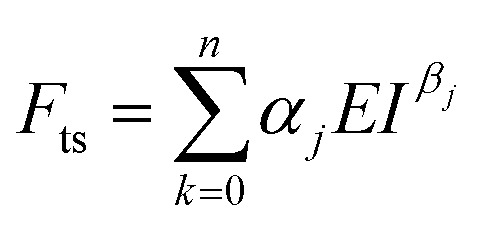


The coefficients *α*_*j*_ and *β*_*j*_ depend on the geometry of the tip and on the finite-thickness of the material. In general, it is more convenient to write eqn (12) as the sum of the force exerted on a semi-infinite system with the same mechanical properties plus a correction term,

13 *F*_ts_ = *F*_semi-infinite_ + *F*_correction_

The bottom-effect correction component gives a series of terms that depend on the indentation, projected area of contact and sample thickness. Compact analytical expressions have been deduced for several axisymmetric tips.^86^ For example, the force applied by a conical tip of half-angle *θ* on an elastic and incompressible material of thickness *h* is given by^86^14a
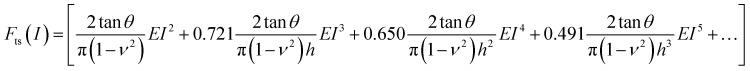
14b
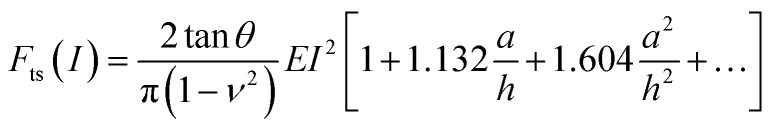
where *a* is the contact radius defined in Table 3. The first term in the above equation coincides with the force deduced by Sneddon (eqn (2)) while the other terms are the bottom-effect corrections. For a paraboloid of radius *R*^86^15a
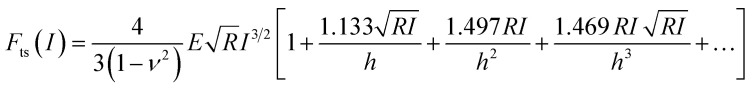
15b
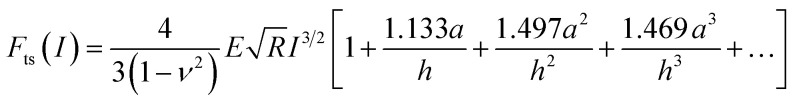


The above equations show that for a given *h*, the bottom stiffness effect is enhanced by increasing the contact area. This result underlines that the use of large tips might enhance the influence of the substrate in the force measured in AFM. The above equations were deduced by assuming that the contact area does not depend on the thickness of the sample.

**Fig. 4:**
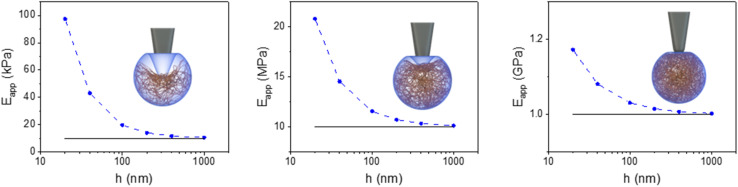
Bottom stiffness effect in nanoparticles. Apparent Young's modulus as a function of the NP diameter for three materials (10 kPa, 10 MPa and 1 GPa). The apparent Young's modulus was obtained by fitting a FDC with the Sneddon model for a paraboloid tip (*R* = 50 nm). The FDCs were generated by applying a maximum force of 0.1 nN for NPs of 10 kPa and 1 nN for the other NPs. The solid line represents the true modulus of the NP.

Fig. 4 illustrates the bottom stiffness effect on nanoparticles (NP) of different sizes (10–1000 nm) and elastic modulus (true values). Apparent modulus values were higher (2 to 10-fold) than true values for small (20–100 nm) and soft (0.01–10 MPa) NPs. The difference was reduced by increasing the diameter and/or the modulus. It fell below 20% for moderately stiff NPs (≥100 MPa). For those NPs, the bottom stiffness effect became negligible for diameters above 200 nm. The above results underline that ignoring the bottom stiffness effect might lead to major errors in the determination of the elastic modulus of nanomaterials.

Analytical bottom-effect correction theories have also been deduced to correct measurements performed in viscoelastic materials.^78,80,89^ Viscoelasticity introduces hysteresis in a force–distance curve. As a consequence, the value of the force, for a given indentation, will depend on whether the tip is approaching or withdrawing from the sample.

Finally, it should be emphasized that, for a given indentation, the increase of the force measured on a finite-thickness material with respect to a semi-infinite material is an unavoidable effect.^11^ Nonetheless, true values of the mechanical parameters might be obtained by fitting the data with the appropriate bottom-effect correction model.

## 5. Nanoscale *versus* macroscale measurements

The value of a mechanical property measured by AFM should not necessarily coincide with the value measured by a macroscopic technique on a sample of the same material. AFM measurements provide values averaged over very small volumes of the material, say from few to thousands of nm^3^. On the other hand, macroscale measurements provide average values over a large volume of the material, typically hundreds of mm^3^.

It is known that mechanical properties of heterogeneous surfaces might be dominated by intrinsic size effects. The existence of stress gradients at the domain boundaries,^96,97^ the role of nanoscale interfaces in controlling the mechanical properties of epoxy nanocomposites^98,99^ or the aforementioned bottom stiffness effects are some illustrative examples.

On the other hand, some materials like nanoparticles (NPs) are intrinsically nanoscale systems. The mechanical properties of NPs have been considered a factor in regulating the interactions of NPs and cells and tissues.^100–102^ In fact, the elastic modulus is considered a relevant factor in the design of drug delivery systems to overcome the biological barriers.^103–105^ AFM nanoindentation modes provide direct measurements of their mechanical properties.

It might be informative to compare nanoscale and macroscale measurements performed on homogeneous materials. It has been shown that AFM, nanoindentation and DMA data were in good agreement when the differences in the experimental protocols were also included in the analysis.^46–48^ For example, similar values for the storage modulus, loss modulus and loss tangent were measured by nanoscale rheology and DMA on several rubbers.^81^ The same conclusion was reached for force volume measurements of the elastic, storage and loss tangent of several polymers and polymers blends^106,107^ and the elastic modulus of silica beads.^108^

## 6. Accuracy, spatial resolution and throughput

### 6.1 Quantitative accuracy

The factors that control the accuracy of a nanomechanical measurement are the theory that describes the mechanical properties of the sample, the theory that transform observables into properties and the calibration of the elements involved in the AFM measurement. Those factors were discussed in detail in ref. 11. We have discussed above bottom-effect corrections. Recently, the sample tilt at the contact area has been identified as a factor that could produce moduli values (*E*_app_) different from true values. Some experiments, theoretical and finite-element simulations have postulated the existence of a correction factor associated with the sample tilt,^109–111^

16 *E*_app_ = *c*_f_*E*

For conical and pyramidal tips, the correction factor is very close to 1 (1.05) for sample tilt angles below 20°. For a spherical tip, the correction factor is about 0.85 (sample tilt angle ≤ 20°).^111^ Interestingly, the correction factor is ≥ 1 for conical tips and ≤ 1 for spherical tips.

### 6.2 Spatial resolution in nanomechanical mapping

The lateral resolution *l*_r_ might be defined as the diameter of the projected area of contact,^2^

17 *l*_r_ = 2*a*

for a paraboloid of radius *R*, the lateral resolution might be approximated by18
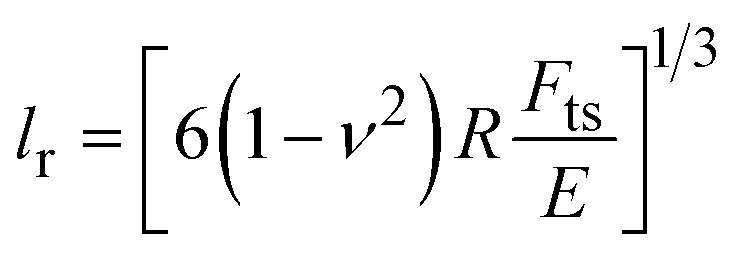
while for a conical tip of half-angle *θ*19
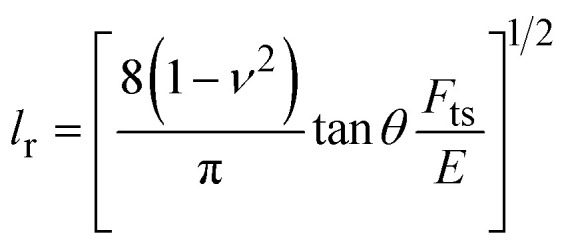


The above expressions are valid for both topography and mechanical property maps.

**Fig. 5:**
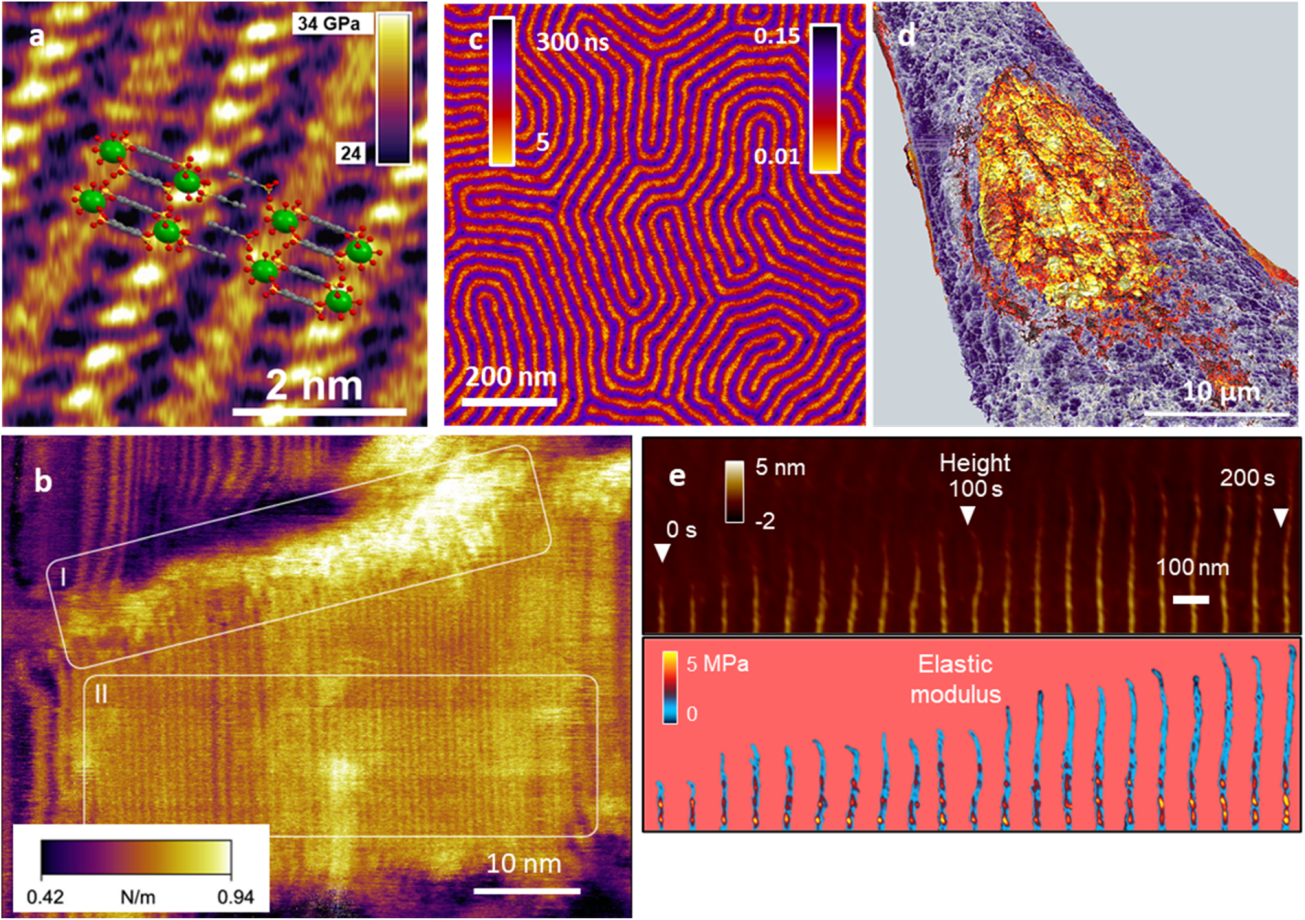
Examples of nanomechanical mapping of surfaces and interfaces. (a) Elastic modulus map of a metal–organic-framework with angstrom-scale resolution (bimodal AFM). Reprinted with permission from ref. 112. Copyright 2017 American Chemical Society. (b) Stiffness map of a polyethylene film (bimodal AFM). The image shows a lamella with disordered end chains at the interface of the crystalline and amorphous phases (region I) and tightly packed polymer chains (region II). Adapted with permission from ref. 113. Copyright 2018 American Chemical Society. (c) Retardation time and loss tangent map of a PS-b-PMMA block co-polymer (bimodal AFM). Adapted with permission from ref. 82. Copyright 2019 Royal Society Chemistry. (d) High-spatial resolution map on a HeLa cell generated by combining topography and elastic modulus data (force volume). The map shows the fine structure of the actin filament network, the local variation of the modulus, and the size and shape of the nucleus. Reprinted with permission from ref. 116. Copyright 2025 Wiley-VCH GmbH. (e) Kymograph of the height and the elastic modulus of a growing collagen nanofibril. The images show the transition from the accretion of collagen precursors from the solution to the formation of a collagen nanofibril (five tropocollagen molecules) with the D-band structure. Imaging rate, 1.12 fps (256 × 256 pixels). Reprinted with permission from ref. 60. Copyright 2021 American Chemical Society.

Several nanomechanical maps showed a spatial resolution in the sub-nanometer range.^112–115^ Angstrom-scale resolution was demonstrated by mapping the elastic response of the different atoms in a metal–organic-framework^112^ (Fig. 5a). True molecular resolution contrast and mechanical property mapping has been provided on several polymer surfaces,^113^ organic semiconductors and organic thin films^114,115^ (Fig. 5b). Viscoelastic property maps have been generated with sub-10 nm spatial resolution. Fig. 5c shows the loss tangent and retardation maps of a block-copolymer.^82^

Fig. 5d shows a force volume map of a live cell. The map was made by combining in each *xy* position, the modulus and cell's height values.^116^ The map resolved the mesh structure of the actin filament network and the size and shape of the nucleus. It also revealed the existence of patches of different scaling modulus values. Those patches were more abundant over the nuclear region. In practical terms, nanomechanical maps might be generated with a lateral spatial resolution comparable to the corresponding topographic image.

### 6.3 Throughput

The throughput of nanomechanical mapping modes is controlled by two independent processes, data acquisition and mechanical property determination.

A key parameter to estimate the throughput in force volume is the value of the frequency *f*_d_ of the signal that modulates the tip-sample distance. Force volume maps generated by using a triangular waveform are limited by the tip's velocity (*v*). First, the equivalent frequency (*f*_d_ = *v*/2*d*_*z*_) has to be significantly lower than the resonance frequency of the *z*-piezo scanner *f*_*pz*_. That frequency might be of a few kHz for *z*-scanners with a *z* range of few microns. Second, the existence of tip's discontinuity at the turning point also limits the imaging rate. Those factors explain throughputs in the tens of minutes range. As an example, a force volume map (40k pixels) acquired at *f*_d_ = 100 Hz might take about 14 min. The use of sinusoidal waveforms (off-resonance) to generate FDCs has reduced the acquisition time.^17,26^ Nonetheless, mechanical actuated (piezo-driven) off-resonance force volume modes are still limited by the value of *f*_*pz*_ (see above). Currently the maximum nominal off-resonance frequency of mechanically actuated systems is about 5 kHz. That limit has been extended to 25 kHz by using photothermal actuation.^117^ However, the effective pixel frequency might be lower because in every pixel the FDCs are averaged over several cycles to obtain reliable values. Photothermal off-resonance excitation has generated a topography and an elastic modulus map of a polymer blend at 1 frame per minute (436 × 436 pixels) which means an effective off-resonance frequency *f*_d_ = 6 kHz.^117^ This result represents an improvement by a factor three over force volume modes based on mechanical actuation.

Nano-DMA and contact resonance modes neither have been designed nor optimized for high throughputs. In nano-DMA the throughput is limited by the tip's velocity during the approach (and retraction) sections and by number of periods needed to get a reliable measurement. For example, a nano-DMA map (64 × 64 pixels) obtained with a modulation frequency of 105 Hz took about 20 minutes.^96^ To generate a 40k pixel map would take more than 3 hours.

Bimodal AFM stands out by achieving genuine high-speed rates. Fig. 5e shows some elastic modulus and loss tangent snapshots of collagen self-assembly processes which were acquired at 1.5 fps (200 × 200 pixels).^60^ The high throughput achieved by bimodal AFM derives from three factors. First, the cantilever-tip system is driven at resonance conditions (hundreds of kHz to few MHz). Second, the use of small oscillation amplitudes (1–10 nm) which prevents the excitation of mechanical resonances in the *xyz* scanners. Third, the use of an amplitude modulation (tapping) feedback for imaging.^2,30^ Those features make bimodal AFM mapping compatible with the technology developed for high-speed AFM.^118^

Bimodal AFM is the only nanomechanical mapping mode that generates simultaneously topography and mechanical property values. The existence of simple analytical expressions which relate observables with mechanical parameters (Section 3) explains this feature. The other nanomechanical modes involve some non-linear least squares fitting processes that increase significantly the time required to generate a nanomechanical map. Conversely, those processes reduce the throughput. Machine learning methods might provide a solution to post-processing issues.^116,119^

## 7. Machine learning

The potential of machine learning methods to improve data acquisition and processing in AFM is emerging. Machine learning applications in AFM have been mostly devoted to improving imaging capabilities for classifying images,^120,121^ correcting imaging errors^122^ or remove noise.^123^

Dielectric property maps of fixed cells were obtained by combining electrostatic force volume data with machine learning.^124^ In the context of AFM-based mechanical spectroscopy, some contributions have explored the capability to predict the forces exerted on a material by using tapping mode, peak force tapping or bimodal AFM.^125–127^ Mechanical property mapping and machine learning were combined to propose a method to identify material components at the nanoscale.^128,129^ Some contributions have compared several regressors to predict the elastic modulus of cells based on force *versus* distance curves^130,131^ or proposed self-organized neural networks to process mechanical properties of cells.^132^ Our group has developed a supervised machine learning regressor for transforming AFM force–distance curves into rheological or viscoelastic properties.^117,119^ The method reduces the computational time required to process a force volume map of a cell made of 2.62 × 10^5^ curves from several hours to minutes (Fig. 5d). The map represented the modulus and the fluidity of a HeLa cell on each point of the cell surface. Indeed, the classification and sorting out of cells by the mechanical phenotype seems an area of great potential.

## 8. Applications

### 8.1 Nanomechanical property mapping

AFM-based nanomechanical mapping modes have been extensively applied to characterize material properties in several fields, disciplines and subdisciplines. Together with the contributions mentioned above we note some recent contributions using nanomechanical mapping modes to address scientific problems of materials science,^133–139^ energy storage,^140–144^ biomedicine,^145–150^ mechanobiology^151–161^ and nanomedicine.^162–165^

The following results illustrate the variety, relevance and novelty of nanomechanical mapping applications. Nanomechanical maps have contributed to clarify the role of ion transport in organic electrochemical semiconductors.^114,140^ Insights on the evolution of the solid electrolyte interphase on battery electrodes were achieved by correlating local topographic and mechanical property features.^142–144^

Biomedical applications are illustrated by the use of nanomechanical maps to characterize cancer cells.^145,146,148,150,161^ The relationship between active intracellular forces and passive viscoelastic properties at the single cell level was also studied.^147^ The correlation between tissue aging and elastic modulus changes was addressed in bone^155^ and in collagen nanoribbons.^156^ Nanomechanical maps have revealed how variations in collagen fibril structure might lead to mechanical susceptibility to damage.^157,158^ AFM-based indentation has elucidated fatigue and other mechanical properties of a virus capsid.^159,160^

Several types of nanoparticles (NP) have been incorporated for drug delivery in pharmaceutics. A variety of studies have shown the relevance of the NP stiffness in regulating different process such as endocytosis, phagocytosis and tumor-targeting delivery.^100–105^ AFM nanoindentation has been applied to quantify the stiffness of NPs in terms of the Young's modulus.^102,103,163–165^ Those measurements were performed without applying bottom-effect corrections. It seems reasonable to assume that in some of those measurements the elastic modulus was overestimated (Fig. 4). Bimodal AFM was used to identify the preferential adsorption sites of iron oxide NPs in liposomes.^162^

The characterization of materials at the nanoscale is enhanced by implementing correlative approaches. Nanomechanical mapping has been combined with transmission electron microscopy,^166^ super resolution microscopy,^167^ confocal microscopy,^168,169^ fluorescence,^170^ infra-red spectroscopy,^171^ ion conductance,^172^ X-ray reflectivity^173^ or traction force microscopy.^147^

AFM-based indentation modes have stimulated the study of viscoelastic properties of polymers, proteins, cells and soft matter in general.^174–181^ To a certain extent, our understanding of viscoelastic properties of nano and mesoscale materials comes from AFM experiments.

**Fig. 6:**
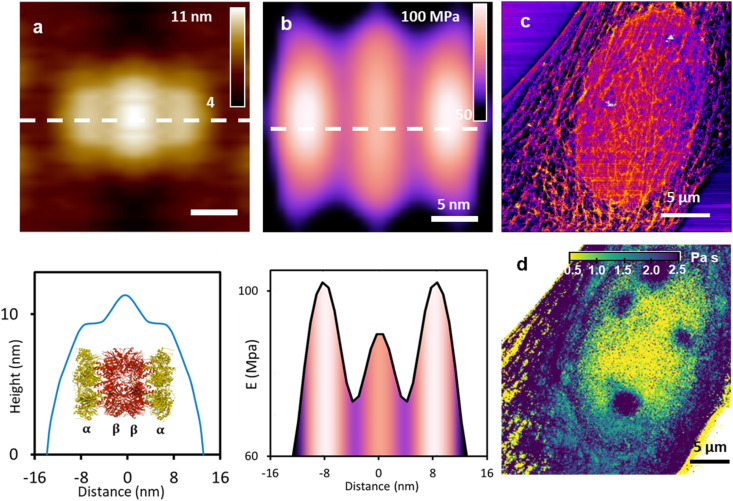
Topography images and mechanical property maps. (a) Topography and elastic modulus map (b) of the 20S proteasome. The bottom panels show the respective cross-sections along the marked lines of the images. The height and elastic modulus cross-sections are anti-correlated. The inset shows a scheme of the 20S proteasome (Protein Data Bank 5L4G). Proteasome images obtained by bimodal AFM. Reprinted with permission from ref. 51. Copyright 2018 Springer Nature. (c). Topography of the actin cytoskeleton; cell depth = 0–100 nm. (d). Viscous coefficient map, cell depth = 1000 nm. The viscous coefficient map shows components of the nucleus that are not resolved in the topographic image (c). Cell images obtained by force volume. Adapted with permission from ref. 182. Copyright 2019 American Chemical Society.

The range of applications, sensitivity, spatial and time resolutions achieved by AFM-based indentation modes are illustrated in Fig. 5 and 6. Viscoelastic behaviour was revealed in a retardation time map of a block co-polymer^82^ (Fig. 5c). The combination of topography and elastic module images improved the contrast of the cortex structure of live cells^117^ (Fig. 5d). Bimodal AFM followed in real-time the evolution of the elastic modulus during the growth of a collagen nanofibril. The images showed the relationship between structure and mechanical stiffness^60^ (Fig. 5f).

Fig. 6 compares topography and mechanical property maps across nano and micrometer scales. The comparison aims to underline the complementarity between topographic images and nanomechanical property maps. Fig. 6a shows height image and Fig. 6b the elastic modulus map of 20S proteasome.^51^ The topography images and the elastic modulus map were generated simultaneously by bimodal AFM. The top panels reveal a protein made of three units (see inset, bottom panel in Fig. 6a). The cross-sections along the dashed lines of the images are shown in the bottom panels. Topography and elastic modulus peaks are anti-correlated. This indicates the absence of cross-talk between morphology and mechanical properties. The height profile showed a symmetric structure that peaked in the middle (11 nm). The Young's modulus profile showed also a symmetric structure but with the maxima were located at the edges of the protein (102 MPa). The differences observed in the elastic modulus values of the sub-units were indicative of their respective functionalities in the protein.^51^

Fig. 6c and d shows two force volume images of a cell, a high-pass height image (Fig. 6c) and a viscosity map (Fig. 6d). The viscosity map revealed subsurface structure of the nucleus while the height image was dominated by the actin cytoskeleton architecture of the cell cortex.^182^

### 8.2 Beyond two-dimensional nanomechanical mapping

It is worth to single out two applications of force volume, nanomechanical tomography and 3D-AFM. Those applications go beyond the conventional definition of nanomechanical mapping.

#### 8.2.1 Nanomechanical tomography

It is a force volume mode which represents the spatial variation of the force as a function of the indentation depth (*F*(*x*, *y*, *z*)). In its first implementation, Kasas and co-workers plotted the Young's modulus at different depths to distinguish regions of different stiffness.^183^ The Young's modulus was obtained by fitting different sections of a FDC. Nanomechanical tomography has been applied to generate spatially-resolved mechanical property maps taken at different sample depths of cells,^182–186^ polymers^187,188^ and solid electrolyte interphase.^143^ It has stimulated also some theoretical studies.^189–191^ The contrast and the spatial resolution to imaging subsurface structures in living cells was improved by fitting the FDCs with a linear viscoelastic model.^182^

**Fig. 7:**
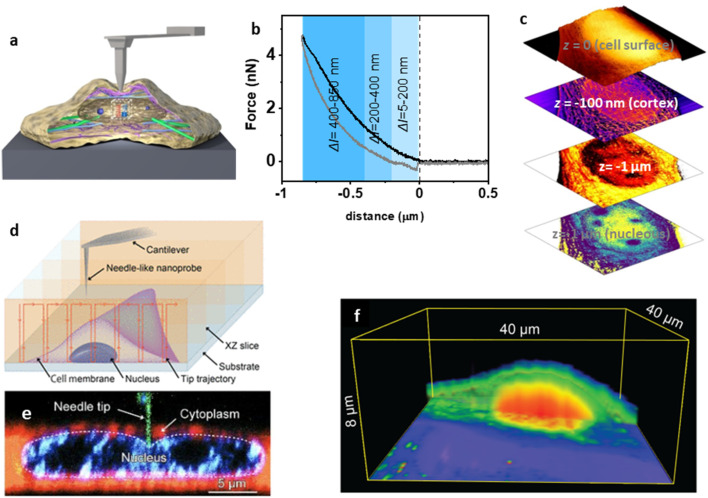
Nanomechanical tomography. (a) Schematic of a tip–cell interface in force volume. (b) Force–distance curve acquired on a cell (NIH 3T3 fibroblast) immersed in buffer. (c) Nanotomography map of a cell. From top to bottom, topography of the cell surface (cell depth = 0 nm); topography of the actin cytoskeleton (cell depth = 0–100 nm); viscous coefficient map (cell depth = 1000 nm). Reprinted with permission from ref. 182. Copyright 2019 American Chemical Society. (d) Schematic of nanoendoscopy-AFM. The tip is repeatedly introduced inside the cell at different lateral positions. (e) Confocal microscopy image of a tip inside the cytosol pressing against the nucleus. The nanoneedle tip, cytoplasm, and nucleus were stained green, red, and blue, respectively. (f) Nanoendoscopy-AFM cell map of a HeLa cell. Panels d and f reprinted with permission from ref. 192 Copyright 2021 AAAS. Panel e reprinted with permission from ref. 193.

Fig. 7a–c shows a force volume configuration for imaging subsurface structures in cells.^182^ The process involved the acquisition of FDCs (retraction and approaching sections) on each point of the cell surface (Fig. 7b). Then, a FDC was separated into different indentation (depth) sections. Each FDC section was processed by using the Kelvin–Voigt linear viscoelastic model. This step gave both a 2D map of the Young's modulus and the viscosity coefficient. Those 2D maps were organized as a function of the depth (mean value within a section) to provide a 3D representation of the cell's structure and mechanical response (Fig. 7c). This method generated a 3D image of a cell made of three layers, the surface topography, a very high spatial resolution image of the cortex with the details of the actin cytoskeleton structure near the plasma membrane and the structure of the nucleus. It was noted that the viscoelasticity coefficient provided higher contrast images of the nucleoli inside nucleus.^182^

Fukuma and co-workers developed another nanomechanical tomography configuration called nanoendoscopy-AFM.^192,193^ This technique repeatedly inserts a nanoneedle tip into the cell to measure FDCs at different *xy* positions (Fig. 7d). Long needle tips are needed to break the cell membrane and penetrate inside the cell (Fig. 7e). A low-resolution volume image of a HeLa cell was obtained by plotting the force as a function of the spatial coordinates. The cell membrane, nucleus, and cytoplasmic regions can be distinguished in the cross section displayed in Fig. 7f.

In the context of subsurface imaging, it has to be noted that contact resonance AFM has been applied to imaging subsurface structures. The experimental set-ups and the theoretical background are far from the ones based on force volume. The interested reader is directed to some recent reviews.^12,13,54,194^

#### 8.2.2 3D-AFM

**Fig. 8:**
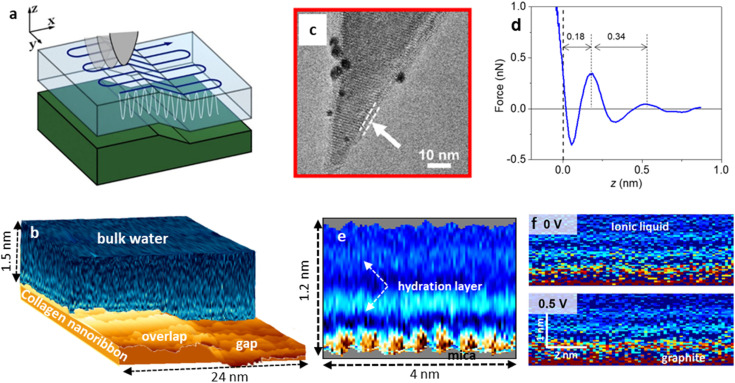
3D-AFM. (a). Scheme of the *xyz* tip displacements in 3D-AFM imaging of solid–liquid interfaces. (b) 3D-AFM volume image of a collagen–water interface. Two hydration layers follow the contours of the collagen nanoribbon. The water covers the whole collagen nanoribbon surface. To facilitate interpretation, the image is divided in two regions, the collagen surface (brown) and the structure of the interfacial water (blue). Based on data from ref. 205. (c) TEM images of hydrophilic silicon-based tip. Arrows and dashed lines highlight the oxidation layer covering the silicon probe. Panel adapted with permission from ref. 202. Copyright 2023 American Chemical Society. (d) Force–distance curve measured on mica–water interface. (e) 2D force (*x*, *z*) map of a 200 mM KCl solution near a mica surface. Adapted with permission from ref. 39. Copyright 2019 Springer Nature. (f) 2D force (*x*, *z*) map of an ionic liquid on a graphite surface measured at two surface potentials. The light stripes show the layering of the ionic liquid molecules. Adapted with permission from ref. 196. Copyright 2020 American Chemical Society.

Three-dimensional AFM has become the most powerful method to characterize solid–liquid interfaces at the molecular scale.^33–43,195–205^ It combines a resonant oscillation of the tip with an off-resonance *z*-displacement. In 3D-AFM the tip is displaced above a solid surface in the *xyz* space by synchronizing the three spatial components of the tip displacement. The tip oscillates at one of its flexural resonances while it explores the solid–liquid interface (Fig. 8a and b). Fig. 8c shows a high resolution transmission electron microscopy image of one of the tips used in 3D-AFM. A force–distance curve is measured at each (*x*,*y*) of the surface (Fig. 8d). Those curves are integrated in a series of *xz* planes, one per each *y* position (Fig. 8e and f). Those planes might be combined to generate a volume map of the interface (Fig. 8b). To achieve molecular resolution, the amplitude of the tip's oscillation must be several times smaller than the molecular diameter of the liquid molecules. For neutral tips, the force measured by the tip near a solid surface is almost proportional to the mass density of the solvent.^206–210^

## 9. Conclusion

This review introduced some key features of the most popular nanomechanical mapping modes which operate by detecting repulsive forces. AFM-based indentation modes provide mechanical property maps of surfaces and interfaces with nanoscale spatial resolution in liquid and ambient pressure environments.

About 30 years ago the emergence of nanotechnology motivated the development of force volume. The first nanomechanical mapping mode was based on the acquisition of force–distance curves on each point of a surface. This breakthrough was followed by the development of parametric modes such as bimodal AFM which enabled nanomechanical mapping at high-speed rates. Those modes together with nano-DMA are shaping our understanding of viscoelasticity at the nanoscale level.

Nanomechanical mapping concepts have been applied to generate 3D images of materials and interfaces. In nanomechanical tomography, 2D mechanical property maps obtained at different depths were organized to reveal the subsurface structure of cells and polymers. On the other hand, 3D-AFM is an advanced realization of force volume imaging that enabled studying solid–liquid interfaces at the molecular level.

Nowadays, a variety of fields and disciplines such as mechanobiology, nanomedicine, energy storage or the development of smart soft materials provide the scientific and technological stimulus to expand, update and refine the capabilities of nanomechanical mapping. We envision a new generation of AFM instruments that will integrate advanced tip functionalization, automatization, machine learning, quantitative accuracy, nanoscale spatial resolution and high-speed capabilities for the mechanical characterization of materials in their native state and environment.

## Author contributions

RG contributed to conceptualization, data analysis, original draft, editing, manuscript preparation, funding acquisition and supervision. JRT contributed to data analysis, editing and manuscript preparation.

## Conflicts of interest

On behalf of all authors, the corresponding author states that there is no conflict of interest.

## Data Availability

All relevant data are within the manuscript and its additional files.

## References

[cit1] Binnig G., Quate C. F., Gerber C. (1986). Atomic Force Microscope. Phys. Rev. Lett..

[cit2] Garcia R. , Amplitude Modulation AFM, Wiley-VCH, 2011

[cit3] Radmacher M., Cleveland J. P., Fritz M., Hansma H. G., Hansma P. K. (1994). Mapping interaction forces with the atomic force microscope. Biophys. J..

[cit4] Heinz W. F., Hoh J. H. (1999). Spatially resolved force spectroscopy of biological surfaces using the atomic force microscope. Trends Biotechnol..

[cit5] Garcia R., Proksch R. (2013). Nanomechanical mapping of soft matter by bimodal force microscopy. Eur. Polym. J..

[cit6] Zhang S., Aslan H., Besenbacher F., Dong M. (2014). Quantitative biomolecular imaging by dynamic nanomechanical mapping. Chem. Soc. Rev..

[cit7] Dufrêne Y. F., Ando T., Garcia R., Alsteens D., Martinez-Martin D., Engel A., Gerber C., Müller D. J. (2017). Imaging Modes of Atomic Force Microscopy for Application in Molecular and Cell Biology. Nat. Nanotechnol..

[cit8] Claesson P. M., Dobryden I., Li G., He Y., Huang H., Thorén P., Haviland D. B. (2017). From force curves to surface nanomechanical properties. Phys. Chem. Chem. Phys..

[cit9] Xu K., Sun W., Shao Y., Wei F., Zhang X., Wang W., Li P. (2018). Recent development of PeakForce Tapping mode atomic force microscopy and its applications on nanoscience. Nanotechnol. Rev..

[cit10] Krieg M., Fläschner G., Alsteens D., Gaub B. M., Roos W. H., Wuite G. J. L., Gaub H. E., Gerber C., Dufrêne Y. F., Müller D. J. (2019). Atomic force microscopy-based mechanobiology. Nat. Rev. Phys..

[cit11] Garcia R. (2020). Nanomechanical mapping of soft materials with the atomic force microscope: methods, theory and applications. Chem. Soc. Rev..

[cit12] Ma C., Arnold W. (2020). Nanoscale ultrasonic subsurface imaging with atomic force microscopy. J. Appl. Phys..

[cit13] Stan G., King S. W. (2020). Atomic force microscopy for nanoscale mechanical property characterization. J. Vac. Sci. Technol., B:Nanotechnol. Microelectron.:Mater., Process., Meas., Phenom..

[cit14] Müller D. J., Dumitru A. C., Lo Giudice C., Gaub H. E., Hinterdorfer P., Hummer G., De Yoreo J. J., Dufrêne Y. F., Alsteens D. (2021). Atomic Force Microscopy-Based Force Spectroscopy and Multiparametric Imaging of Biomolecular and Cellular Systems. Chem. Rev..

[cit15] Collinson D. W., Sheridan R. J., Palmeri M. J., Brinson L. C. (2021). Best practices and recommendations for accurate nanomechanical characterization of heterogeneous polymer systems with atomic force microscopy. Prog. Polym. Sci..

[cit16] Krüger S., Krüger D., Janshoff A. (2004). Scanning Force Microscopy Based Rapid Force Curve Acquisition on Supported Lipid Bilayers: Experiments and Simulations Using Pulsed Force Mode. ChemPhysChem.

[cit17] Pittenger B. , Erina N. and Su C., Quantitative Mechanical Property Mapping at the Nanoscale with PeakForce QNM, Bruker Application note, 2010, 128, 10.13140/RG.2.1.4463.8246

[cit18] Herruzo E. T., Perrino A. P., Garcia R. (2014). Fast nanomechanical spectroscopy of soft matter. Nat. Commun..

[cit19] Dokukin M. E., Sokolov I. (2017). Nanoscale compositional mapping of cells, tissues, and polymers with ringing
mode of atomic force microscopy. Sci. Rep..

[cit20] Efremov Y. M., Cartagena-Rivera A. X., Athamneh A. I. M., Suter D. M., Raman A. (2018). Mapping heterogeneity of cellular mechanics by multi-harmonic atomic force microscopy. Nat. Protoc..

[cit21] Gisbert V. G., Garcia R. (2023). Fast and high-resolution mapping of van der Waals forces of 2D materials interfaces with bimodal AFM. Nanoscale.

[cit22] Santos S., Elsherbiny L., Lai C., Askar K., Gadelrab K., Chiesa M. (2024). Automatic Generation of Contrast Maps in Terms of van der Waals Material Properties in Bimodal AFM. J. Phys. Chem. C.

[cit23] Pfreundschuh M., Martinez-Martin D., Mulvihill E., Wegmann S., Muller D. J. (2014). Multiparametric high-resolution imaging of native proteins by force-distance curve-based AFM. Nat. Protoc..

[cit24] Nievergelt A. P., Banterle N., Andany S. H., Gönczy P., Fantner G. E. (2018). High-speed photothermal off-resonance atomic force microscopy reveals assembly routes of centriolar scaffold protein SAS-6. Nat. Nanotechnol..

[cit25] Meng X., Zhang H., Song J., Fan X., Sun L., Xie H. (2017). Broad modulus range nanomechanical mapping by magnetic-drive soft probes. Nat. Commun..

[cit26] Li P., Shao Y., Xu K., Liu X. (2023). High-speed multiparametric imaging through off-resonance tapping AFM with active probe. Ultramicroscopy.

[cit27] Ratcliff G. C., Erie D. A., Superfine R. (1998). Photothermal modulation for oscillating mode atomic force microscopy in solution. Appl. Phys. Lett..

[cit28] Ramos D., Tamayo J., Mertens J., Calleja M. (2006). Photothermal excitation of microcantilevers in liquids. J. Appl. Phys..

[cit29] Labuda A., Kobayashi K., Kiracofe D., Suzuki K., Grütter P. H., Yamada H. (2011). Comparison of photothermal and piezoacoustic excitation methods for frequency and phase modulation atomic force microscopy in liquid environments. AIP Adv..

[cit30] García R., San Paulo A. (1999). Attractive and repulsive tip-sample interaction regimes in tapping-mode atomic force microscopy. Phys. Rev. B:Condens. Matter Mater. Phys..

[cit31] Hölscher H. (2006). Quantitative measurement of tip-sample interactions in amplitude modulation atomic force microscopy. Appl. Phys. Lett..

[cit32] Payam A. F., Martin-Jimenez D., Garcia R. (2015). Force reconstruction from tapping mode force microscopy experiments. Nanotechnol.

[cit33] Fukuma T., Garcia R. (2018). Atomic- and Molecular-Resolution Mapping of Solid–Liquid Interfaces by 3D Atomic Force Microscopy. ACS Nano.

[cit34] Garcia R., Perez R. (2002). Dynamic atomic force microscopy methods. Surf. Sci. Rep..

[cit35] Fukuma T., Ueda Y., Yoshioka S., Asakawa H. (2010). Atomic-Scale Distribution of Water Molecules at the Mica–Water Interface Visualized by Three-Dimensional Scanning Force Microscopy. Phys. Rev. Lett..

[cit36] Kimura K., Ido S., Oyabu N., Kobayashi K., Hirata Y., Imai T., Yamada H. (2010). Visualizing water molecule distribution by atomic force microscopy. J. Chem. Phys..

[cit37] Herruzo E. T., Asakawa H., Fukuma T., Garcia R. (2013). Three-dimensional quantitative force maps in liquid with 10 piconewton, angstrom and sub-minute resolutions. Nanoscale.

[cit38] Martin-Jimenez D., Chacon E., Tarazona P., Garcia R. (2016). Atomically resolved three-dimensional structures of electrolyte aqueous solutions near a solid surface. Nat. Commun..

[cit39] Uhlig M. R., Martin-Jimenez D., Garcia R. (2019). Atomic-scale mapping of hydrophobic layers on graphene and few-layer MoS2 and WSe2 in water. Nat. Commun..

[cit40] Uhlig M. R., Benaglia S., Thakkar R., Comer J., Garcia R. (2021). Atomically resolved interfacial water structures on crystalline hydrophilic and hydrophobic surfaces. Nanoscale.

[cit41] Li Z., Liu Q., Zhang D., Wang Y., Zhang Y., Li Q., Dong M. (2022). Probing the hydration friction of ionic interfaces at the atomic scale. Nanoscale Horiz..

[cit42] Tang Z., Lin S., Wang Z. L. (2024). Unveiling Contact-Electrification Effect on Interfacial Water Oscillation. Adv. Mater..

[cit43] Ai Q., Bonagiri L. K. S., Farokh Payam A., Aluru N. R., Zhang Y. (2025). Toward Quantitative Interpretation of 3D Atomic Force Microscopy at Solid–Liquid Interfaces. J. Phys. Chem. C.

[cit44] Cohen S. R., Kalfon-Cohen E. (2013). Dynamic nanoindentation by instrumented nanoindentation and force microscopy: a comparative review. Beilstein J. Nanotechnol..

[cit45] Dokukin M., Sokolov I. (2015). High-resolution high-speed dynamic mechanical spectroscopy of cells and other soft materials with the help of atomic force microscopy. Sci. Rep..

[cit46] Pittenger B., Osechinskiy S., Yablon D., Mueller T. (2019). Nanoscale DMA with the Atomic Force Microscope: A New Method for Measuring Viscoelastic Properties of Nanostructured Polymer Materials. JOM.

[cit47] Kolluru P. V., Eaton M. D., Collinson D. W., Cheng X., Delgado D. E., Shull K. R., Brinson L. C. (2018). AFM-based Dynamic Scanning Indentation (DSI) Method for Fast, High-resolution Spatial Mapping of Local Viscoelastic Properties in Soft Materials. Macromolecules.

[cit48] Piacenti A. R., Adam C., Hawkins N., Wagner R., Seifert J., Taniguchi Y., Proksch R., Contera S. (2024). Nanoscale Rheology: Dynamic Mechanical Analysis over a Broad and Continuous Frequency Range Using Photothermal Actuation Atomic Force Microscopy. Macromolecules.

[cit49] Gisbert V. G., Espinosa F. M., Sanchez J. G., Serrano M. C., Garcia R. (2024). Nanorheology and Nanoindentation Revealed a Softening and an Increased Viscous Fluidity of Adherent Mammalian Cells upon Increasing the Frequency. Small.

[cit50] Menard K. P. and Menard N., Dynamic Mechanical Analysis, CRC Press, 2008

[cit51] Benaglia S., Gisbert V. G., Perrino A. P., Amo C. A., Garcia R. (2018). Fast and high-resolution mapping of elastic properties of biomolecules and polymers with bimodal AFM. Nat. Protoc..

[cit52] Killgore J. P., Del Rio F. W. (2018). Contact resonance force microscopy for viscoelastic property measurements: from fundamentals to state-of-the-art applications. Macromolecules.

[cit53] Stan G., Ciobanu C. V., King S. W. (2022). Resolving the Subsurface Structure and Elastic Modulus of Layered Films *via* Contact Resonance Atomic Force Microscopy. ACS Appl. Mater. Interfaces.

[cit54] Farokh Payam A., Passian A. (2023). Imaging beyond the surface region: Probing hidden materials *via* atomic force microscopy. Sci. Adv..

[cit55] Dokukin M. E., Sokolov I. (2012). Quantitative Mapping of the Elastic Modulus of Soft Materials with HarmoniX and PeakForce QNM AFM Modes. Langmuir.

[cit56] Cartagena-Rivera A. X., Wang W., Geahlen R. L., Raman A. (2015). Fast, multi-frequency and quantitative nanomechanical mapping of live cells using the atomic force microscope. Sci. Rep..

[cit57] Efremov Y. M., Suter D. M., Timashev P. S., Raman A. (2022). 3D nanomechanical mapping of subcellular and sub-nuclear structures of living cells by multi-harmonic AFM with long-tip microcantilevers. Sci. Rep..

[cit58] Martinez-Martin D., Herruzo E. T., Dietz C., Gomez-Herrero J., Garcia R. (2011). Noninvasive Protein Structural Flexibility Mapping by Bimodal Dynamic Force Microscopy. Phys. Rev. Lett..

[cit59] Labuda A., Kocuń M., Meinhold W., Walters D., Proksch R. (2016). Generalized Hertz model for bimodal nanomechanical mapping. Beilstein J. Nanotechnol..

[cit60] Gisbert V. G., Benaglia S., Uhlig M. R., Proksch R., Garcia R. (2021). High-Speed Nanomechanical Mapping of the Early Stages of Collagen Growth by Bimodal Force Microscopy. ACS Nano.

[cit61] Garcia R., Magerle R., Perez R. (2007). Nanoscale Compositional Mapping with Gentle Forces. Nat. Mater..

[cit62] Tamayo J., Garcia R. (1996). Deformation, contact time and phase contrast in tapping mode scanning force microscopy. Langmuir.

[cit63] Korolkov V. V., Summerfield A., Murphy A., Amabilino D. B., Watanabe K., Taniguchi T., Beton P. H. (2019). Ultra-high resolution imaging of thin films and single strands of polythiophene using atomic force microscopy. Nat. Commun..

[cit64] Lavini F., Cellini F., Rejhon M., Kunc J., Berger C., de Heer W., Riedo E. (2020). Atomic force microscopy phase imaging of epitaxial graphene films. JPhys Mater..

[cit65] Chiodini S., Kerfoot J., Venturi G., Mignuzzi S., Alexeev E. M., Teixeira Rosa B., Tongay S., Taniguchi T., Watanabe K., Ferrari A. C., Ambrosio A. (2022). Moiré Modulation of Van Der Waals Potential in Twisted Hexagonal Boron Nitride. ACS Nano.

[cit66] Murphy J. G., Raybin J. G., Ansay G. E., Sibener S. J. (2023). Spatiotemporal Mapping of Hole Nucleation and Growth during Block Copolymer Terracing with High-Speed Atomic Force Microscopy. ACS Nano.

[cit67] Garcia R., Gómez C. J., Martinez N. F., Patil S., Dietz C., Magerle R. (2006). Identification of Nanoscale Dissipation Processes by Dynamic Atomic Force Microscopy. Phys. Rev. Lett..

[cit68] Santos S., Gadelrab K., Lai C., Olukan T., Font J., Barcons V., Verdaguer A., Chiesa M. (2021). Advances in dynamic AFM: From nanoscale energy dissipation to material properties in the nanoscale. J. Appl. Phys..

[cit69] Preiner J., Losilla N. S., Ebner A., Annibale P., Biscarini F., Garcia R., Hinterdorfer P. (2009). Imaging and Detection of Single Molecule Recognition Events on Organic Semiconductor Surfaces. Nano Lett..

[cit70] Dumitru A. C., Stommen A., Koelher M., Cloos A. S., Yang J., Leclercqz A., Tyteca D., Alsteens D. (2021). Probing PIEZO1 localization upon activation using high-resolution atomic force and confocal microscopy. Nano Lett..

[cit71] Sader J. E., Sanelli J. A., Adamson B. D., Monty J. P., Wei X., Crawford S. A., Friend J. R., Marusic I., Mulvaney P., Bieske E. J. (2012). Spring constant calibration of atomic force microscope cantilevers of arbitrary shape. Rev. Sci. Instrum..

[cit72] Labuda A., Kocun M., Lysy M., Walsh T., Meinhold J., Proksch T., Meinhold W., Anderson C., Proksch R. (2016). Calibration of higher eigenmodes of cantilevers. Rev. Sci. Instrum..

[cit73] Garcia P. D., Guerrero C. R., Garcia R. (2017). Time-resolved nanomechanics of a single cell under the depolymerization of the cytoskeleton. Nanoscale.

[cit74] Tschoegl N. W. , The Phenomenological Theory of Linear Viscoelastic Behavior: an Introduction, Springer, Berlin Heidelberg, Berlin; Heidelberg, 1989

[cit75] Bonfanti A., Kaplan J. L., Charras G., Kabla A. (2020). Fractional viscoelastic models for powerlaw materials. Soft Matter.

[cit76] Niu T., Cao G. (2014). Power-law rheology characterization of biological cell properties under AFM indentation measurement. RSC Adv..

[cit77] Efremov Y. M., Okajima T., Raman A. (2019). Measuring viscoelasticity of soft biological samples using atomic force microscopy. Soft Matter.

[cit78] Garcia P. D., Guerrero C. R., Garcia R. (2020). Nanorheology of living cells measured by AFM-based force-distance curves. Nanoscale.

[cit79] Sanchez J. G., Espinosa F. M., Miguez R., Garcia R. (2021). The viscoelasticity of adherent cells follows a single power-law with distinct local variations within a single cell and across cell lines. Nanoscale.

[cit80] Garcia P. D., Garcia R. (2018). Determination of the viscoelastic properties of a single cell cultured on a rigid support by force microscopy. Nanoscale.

[cit81] Proksch R., Kocun M., Hurley D., Viani M., Labuda A., Meinhold W., Bemis J. (2016). Practical loss tangent imaging with amplitude-modulated atomic force microscopy. J. Appl. Phys..

[cit82] Benaglia S., Amo C. A., Garcia R. (2019). Fast, Quantitative and High-Resolution Mapping of Viscoelastic Properties with Bimodal AFM. Nanoscale.

[cit83] Rosenhek-Goldian I., Cohen S. R. (2023). Some considerations in nanoindentation measurement and analysis by atomic force microscopy. J. Vac. Sci. Technol., A.

[cit84] Sneddon I. N. (1965). The relation between load and penetration in the axisymmetric Boussinesq problem for a punch of arbitrary profile. Int. J. Eng. Sci..

[cit85] Dimitriadis E. K., Horkay F., Maresca J., Kachar B., Chadwick R. S. (2002). Determination of Elastic Moduli of Thin Layers of Soft Material Using the Atomic Force Microscope. Biophys. J..

[cit86] D Garcia P., Garcia R. (2018). Determination of the Elastic Moduli of a Single Cell Cultured on a Rigid Support by Force Microscopy. Biophys. J..

[cit87] Doss B. L., Rahmani Eliato K., Lin K., Ros R. (2019). Quantitative mechanical analysis of indentations on layered, soft elastic materials. Soft Matter.

[cit88] Costa D. F. S., de Araújo J. L. B., Oliveira C. L. N., de Sousa J. S. (2022). Nanoindentation in finite thickness viscoelastic materials. J. Appl. Phys..

[cit89] Cuenot S., Fillaudeau A., Briolay T., Fresquet J., Blanquart C., Ishow E., Zykwinska A. (2025). Poroelastic and viscoelastic properties of soft materials determined from AFM force relaxation and force-distance curves. J. Mech. Behav. Biomed. Mater..

[cit90] Hermanowicz P. (2021). Determination of Young's modulus of samples of arbitrary thickness from force distance curves: numerical investigations and simple approximate formulae. Int. J. Mech. Sci..

[cit91] Argatov I., Jin X. (2024). Self-consistent approximations for the frictionless paraboloidal and conical depth-sensing indentation: The generalized bottom effect. Int. J. Solids Struct..

[cit92] Chiodini S., Ruiz-Rincón S., Garcia P. D., Martin S., Kettelhoit K., Armenia I., Werz D. B., Cea P. (2020). Bottom Effect in Atomic Force Microscopy Nanomechanics. Small.

[cit93] Gisbert V. G., Garcia R. (2021). Accurate wide-modulus-range nanomechanical mapping of ultrathin interfaces with bimodal atomic force microscopy. ACS Nano.

[cit94] Pérez-Domínguez S., Kulkarni S. G., Pabijan J., Gnanachandran K., Holuigue H., Eroles M., Lorenc E., Berardi M., Antonovaite N., Marini M. L., Lopez Alonso J., Redonto-Morata L., Dupres V., Janel S., Acharya S., Otero J., Navajas D., Bielawski K., Schillers H., Lafont F., Rico F., Podestà A., Radmacher M., Lekka M. g. (2023). Reliable, standardized measurements for cell mechanical properties. Nanoscale.

[cit95] Moura A. L. D., Tejedor J. R., Espinosa F. M., Dominguez L. A., de Sousa J. S., Garcia R. (2025). Evidence of the bottom stiffness effect on atomic force microscopy-based cell mechanobiology. Nanoscale.

[cit96] Askes H., Aifantis E. C. (2011). Gradient elasticity in statics and dynamics: An overview of formulations, length scale identification procedures, finite element implementations and new results. Int. J. Solids Struct..

[cit97] Nguyen H. K., Pittenger B., Nakajima K. (2024). Mapping the Nanoscale Heterogeneous Responses in the Dynamic Acceleration of Deformed Polymer Glasses. Nano Lett..

[cit98] Nguyen H. K., Aoki M., Liang X., Yamamoto S., Tanaka K., Nakajima K. (2021). Local Mechanical Properties of Heterogeneous Nanostructures Developed in a Cured Epoxy Network: Implications for Innovative Adhesion Technology. ACS Appl. Nano Mater..

[cit99] Nguyen H. K., Shundo A., Ito M., Pittenger B., Yamamoto S., Tanaka K., Nakajima K. (2023). Insights into Mechanical Dynamics of Nanoscale Interfaces in Epoxy Composites Using Nanorheology Atomic Force Microscopy. ACS Appl. Mater. Interfaces.

[cit100] Anselmo A. C., Zhang M., Kumar S., Vogus D. R., Menegatti S., Helgeson M. E., Mitragotri S. (2015). Elasticity of Nanoparticles Influences Their Blood Circulation, Phagocytosis, Endocytosis, and Targeting. ACS Nano.

[cit101] Hui Y., Yi X., Hou F., Wibowo D., Zhang F., Zhao D., Gao H., Zhao C. (2019). Role of Nanoparticle Mechanical Properties in Cancer Drug Delivery. ACS Nano.

[cit102] Nie D., Liu C., Yu M., Jiang X., Wang N., Gan Y. (2022). Elasticity regulates nanomaterial transport as delivery vehicles: Design, characterization, mechanisms and state of the art. Biomaterials.

[cit103] Li Z., Zhu Y., Zeng H., Wang C., Xu C., Wang Q., Wang H., Li S., Chen J., Xiao C., Yang X., Li Z. (2023). Mechano-boosting nanomedicine antitumour efficacy by blocking the reticuloendothelial system with stiff nanogels. Nat. Commun..

[cit104] Li Z., Xiao C., Yang X., Li Z. (2025). Progress in the mechanical properties of nanoparticles for tumor-targeting delivery. Chem. Soc. Rev..

[cit105] Hui Y., Liu Y., Yang G., Weng Y., Hou F., Wang X., Fang S., Gao H., Zhao C. (2025). Critical Role of Nanomaterial Mechanical Properties in Drug Delivery, Nanovaccines and Beyond. Adv. Mater..

[cit106] Young T. J., Monclus M. A., Burnett T. L., Broughton W. R., Ogin S. L., Smith P. A. (2011). The use of the PeakForceTM quantitative nanomechanical mapping AFM-based method for high-resolution Young's modulus measurement of polymers. Meas. Sci. Technol..

[cit107] Griepentrog M., Kramer G., Cappella B. (2013). Comparison of nanoindentation and AFM methods for the determination of mechanical properties of polymers. Polym. Test..

[cit108] Germanicus R. C., Mercier D., Agrebi F., Fèbvre M., Mariolle D., Descamps P., Leclère P. (2020). Quantitative mapping of high modulus materials at the nanoscale: comparative study between atomic force microscopy and nanoindentation. J. Microsc..

[cit109] Fujii Y., Okajima T. (2019). Calibrating the Young's modulus of soft materials with surface tilt angle measured by atomic force microscopy. AIP Adv..

[cit110] Chiodini S., Borbone F., Oscurato S. L., Garcia P. D., Ambrosio A. (2024). Light-induced modulation of viscoelastic properties in azobenzene polymers. Nanophotonics.

[cit111] Ahmine A. N., Bdiri M., Fereol S., Fodil R. (2024). A comprehensive study of AFM stiffness measurements on inclined surfaces: theoretical, numerical, and experimental evaluation using a Hertz approach. Sci. Rep..

[cit112] Amo C. A., Perrino A. P., Payam A. F., Garcia R. (2017). Mapping Elastic Properties of Heterogeneous Materials in Liquid with Angstrom-Scale Resolution. ACS Nano.

[cit113] Kocun M., Labuda A., Meinhold W., Revenko I., Proksch R. (2017). Fast, High Resolution, and Wide Modulus Range Nanomechanical Mapping with Bimodal Tapping Mode. ACS Nano.

[cit114] Benaglia S., Drakopoulou S., Biscarini F., Garcia R. (2022). In operando nanomechanical mapping of PEDOT:PSS thin films in electrolyte solutions with bimodal AFM. Nanoscale.

[cit115] Arilla R., Barrena E., Ocal C., Martin-Jimenez D. (2025). Bimodal Atomic Force Microscopy with a Torsional Eigenmode for Highly Accurate Imaging of Grain Orientation in Organic Thin Films. Nano Lett..

[cit116] Tejedor J. R., Garcia R. (2025). High-Throughput Nanorheology of Living Cells Powered by Supervised Machine Learning. Adv. Intell. Syst..

[cit117] Gunstheimer H. , Fläschner G., Adams J. D., Hölscher H. and Hoogenboom B. W., High-speed quantitative nanomechanical mapping by photothermal off-resonance atomic force microscopy, arXiv, 2025, 2506.16226 10.1002/smll.202507640 PMC12508714 40838563

[cit118] Ando T. (2012). High-speed atomic force microscopy coming of age. Nanotechnol.

[cit119] Garcia R. and Tejedor J., PCT/EP2024/082497, European Patent Office, 2012

[cit120] Azuri I., Rosenhek-Goldian I., Regev-Rudzki N., Fantner G., Cohen S. R. (2021). The role of convolutional neural networks in scanning probe microscopy: a review. Beilstein J. Nanotechnol..

[cit121] Ziatdinov M., Zhang S., Dollar O., Pfaendtner J., Mundy C. J., Li X., Pyles H., Baker D., De Yoreo J. J., Kalinin S. V. (2021). Quantifying the Dynamics of Protein Self-Organization Using Deep Learning Analysis of Atomic Force Microscopy Data. Nano Lett..

[cit122] Bonagiri L. K. S., Wang Z., Zhou S., Zhang Y. (2024). Precise Surface Profiling at the Nanoscale Enabled by Deep Learning. Nano Lett..

[cit123] Gelman S., Rosenhek-Goldian I., Kampf N., Patočka M., Rios M., Penedo M., Fantner G., Beker A., Cohen S. R., Azuri I. (2025). Deep learning for enhancement of low-resolution and noisy scanning probe microscopy images. Beilstein J. Nanotechnol..

[cit124] Checa M., Millan-Solsona R., Mares A. G., Pujals S., Gomila G. (2021). Fast Label-Free Nanoscale Composition Mapping of Eukaryotic Cells *Via* Scanning Dielectric Force Volume Microscopy and Machine Learning. Small Methods.

[cit125] Chandrashekar A., Belardinelli P., Bessa M. A., Staufer U., Alijani F. (2022). Quantifying nanoscale forces using machine learning in dynamic atomic force microscopy. Nanoscale Adv..

[cit126] Rajabifar B., Meyers G. F., Wagner R., Raman A. (2022). Machine Learning Approach to Characterize the Adhesive and Mechanical Properties of Soft Polymers Using PeakForce Tapping AFM. Macromolecules.

[cit127] Elsherbiny L., Santos S., Gadelrab K., Olukan T., Font J., Barcons V., Chiesa M. (2023). Machine learning assisted multifrequency AFM: Force model prediction. Appl. Phys. Lett..

[cit128] Lai C., Santos S., Chiesa M. (2019). Machine learning assisted quantification of graphitic surfaces exposure to defined environments. Appl. Phys. Lett..

[cit129] Petrov M., Canena D., Kulachenkov N., Kumar N., Nickmilder P., Leclère P., Sokolov I. (2024). Mechanical spectroscopy of materials using atomic force microscopy (AFM-MS). Mater. Today.

[cit130] Nguyen L. T. P., Liu B. H. (2022). Machine learning approach for reducing uncertainty in AFM nanomechanical measurements through selection of appropriate contact model. Eur. J. Mech. A/Solids.

[cit131] Nguyen L. T. P., Liu B. H. (2022). Machine learning framework for determination of elastic modulus without contact model fitting. Int. J. Solids Struct..

[cit132] Weber A., Vivanco M. D. M., Toca-Herrera J. L. (2023). Application of self-organizing maps to AFM-based viscoelastic characterization of breast cancer cell mechanics. Sci. Rep..

[cit133] Ganjian M., Angeloni L., Mirzaali M. J., Modaresifar K., Hagen C. W., Ghatkesar M. K., Hagedoorn P., Fratila-Apachitei L. E., Zadpoor A. A. (2020). Quantitative mechanics of 3D printed nanopillars interacting with bacterial cells. Nanoscale.

[cit134] Angeloni L., Ganjian M., Nouri-Goushki M., Mirzaali M. J., Hagen C. W., Zadpoor A. A., Fratila-Apachitei L. E., Ghatkesar M. K. (2021). Mechanical characterization of nanopillars by atomic force microscopy. Addit. Manuf..

[cit135] Wychowaniec J. K., Moffat J., Saiani A. (2021). Quantitative nanomechanical properties evaluation of a family of β-sheet peptide fibres using rapid bimodal AFM. J. Mech. Behav. Biomed. Mater..

[cit136] Zhang S., Weng Y., Ma C. (2021). Quantitative Nanomechanical Mapping of Polyolefin Elastomer at Nanoscale with Atomic Force Microscopy. Nanoscale Res. Lett..

[cit137] Höppener C., Elter J. K., Schacher F. H., Deckert V. (2023). Inside Block Copolymer Micelles—Tracing Interfacial Influences on Crosslinking Efficiency in Nanoscale Confined Spaces. Small.

[cit138] Lou Z., Zhang Y., Li Y., Xu L. (2023). Study on microscopic physical and chemical properties of biomass materials by AFM. J. Mater. Res. Technol..

[cit139] Chiodini S., De Oliveira M., Borbone F., Moujdi S., Ambrosio A. (2025). Nanomechanical properties of self-structured azopolymer defects [Invited]. Opt. Mater. Express.

[cit140] Giridharagopal R., Flagg L. Q., Harrison J. S., Ziffer M. E., Onorato J., Luscombe C. K., Ginger D. (2017). Electrochemical strain microscopy probes morphology-induced variations in ion uptake and performance in organic electrochemical transistors. Nat. Mater..

[cit141] Zhang Z., Said S., Smith K., Jervis R., Howard C. A., Shearing P. R., Brett D. J. L., Miller T. S. (2021). Characterizing Batteries by *In Situ* Electrochemical Atomic Force Microscopy: A Critical Review. Adv. Energy Mater..

[cit142] Yang P., Bi Z., Shang Y., Chen K., Liang Y., Li X., Shang G. (2021). Bimodal AFM-Based Nanocharacterization of Cycling-Induced Topographic and Mechanical Evolutions of LiMn2O4 Cathode Films. Langmuir.

[cit143] Chen Y., Wu W., Gonzalez-Munoz S., Forcieri L., Wells C., Jarvis S. P., Wu F., Young R., Dey A., Isaacs M., Nagarathinam M., Palgrave R. G., Tapia-Ruiz N., Kolosov O. V. (2023). Nanoarchitecture factors of solid electrolyte interphase formation *via* 3D nano-rheology microscopy and surface force-distance spectroscopy. Nat. Commun..

[cit144] Putra R. P., Matsushita K., Ohnishi T., Masuda T. (2024). Operando Nanomechanical Mapping of Amorphous Silicon Thin Film Electrodes in All-Solid-State Lithium-Ion Battery Configuration during Electrochemical Lithiation and Delithiation. J. Phys. Chem. Lett..

[cit145] Calzado-Martín A., Encinar M., Tamayo J., Calleja M., San Paulo A. (2016). Effect of Actin Organization on the Stiffness of Living Breast Cancer Cells Revealed by Peak-Force Modulation Atomic Force Microscopy. ACS Nano.

[cit146] Schierbaum N., Rheinlaender J., Schäffer T. E. (2017). Viscoelastic properties of normal and cancerous human breast cells are afffected by contact to adjacent cells. Acta Biomater..

[cit147] Schierbaum N., Rheinlaender J., Schaffer T. E. (2019). Combined atomic force microscopy (AFM) and traction force microscopy (TFM) reveals a correlation between viscoelastic material properties and contractile prestress of living cells. Soft Matter.

[cit148] Zhou G., Zhang B., Tang G., Yu X., Galluzzi M. (2021). Cells nanomechanics by atomic force microscopy: focus on interactions at nanoscale. Adv. Phys.:X.

[cit149] Eroles M., Lopez-Alonso J., Ortega A., Boudier T., Gharzeddine K., Lafont F., Franz C. M., Millet A., Valotteau C., Rico F. (2023). Coupled mechanical mapping and interference contrast microscopy reveal viscoelastic and adhesion hallmarks of monocyte differentiation into macrophages. Nanoscale.

[cit150] Kontomaris S. V., Malamou A., Stylianou A. (2025). The Young's Modulus as a Mechanical Biomarker in AFM Experiments: A Tool for Cancer Diagnosis and Treatment Monitoring. Sensors.

[cit151] Li M., Xi N., Wang Y., Liu L. (2019). Advances in atomic force microscopy for single-cell analysis. Nano Res..

[cit152] Mandriota N., Friedsam C., Jones-Molina J. A., Tatem K. V., Ingber D. E., Sahin O. (2019). Cellular nanoscale stiffness patterns governed by intracellular forces. Nat. Mater..

[cit153] Dumitru A. C., Koehler M. (2023). Recent advances in the application of atomic force microscopy to structural biology. J. Struct. Biol..

[cit154] Sitthisang S., Hou X., Treetong A., Xu X., Liu W., He C., Sae-Ueng U., Yodmuang S. (2024). Nanomechanical mapping of PLA hydroxyapatite composite scaffolds links surface homogeneity to stem cell differentiation. Sci. Rep..

[cit155] Kwon J., Cho H. (2023). Nanomechanical Characterization of Bone Quality Depending on Tissue Age *via* Bimodal Atomic Force Microscopy. Nanomanuf. Metrol..

[cit156] Garcia-Sacristan C., Garcia R. (2025). Time-lapsed nanoscale maps of the elastic modulus of collagen during cross-linking by bimodal AFM. Nanoscale.

[cit157] Baldwin S. J., Sampson J., Peacock C. J., Martin M. L., Veres S. P., Lee J. M., Kreplak L. (2020). A new longitudinal variation in the structure of collagen fibrils and its relationship to locations of mechanical damage susceptibility. J. Mech. Behav. Biomed. Mater..

[cit158] Asgari M., Latifi N., Giovanniello F., Espinosa H. D., Amabili M. (2022). Revealing Layer-Specific Ultrastructure and Nanomechanics of Fibrillar Collagen in Human Aorta *via* Atomic Force Microscopy Testing: Implications on Tissue Mechanics at Macroscopic Scale. Adv. NanoBiomed Res..

[cit159] Bruinsma R. F., Wuite G. J. L., Roos W. H. (2021). Physics of viral dynamics. Nat. Rev. Phys..

[cit160] Cantero M., Cvirkaite-Krupovic V., Krupovic M., de Pablo P. J. (2023). Mechanical tomography of an archaeal lemon-shaped virus reveals membrane-like fluidity of the capsid and liquid nucleoprotein cargo. Proc. Natl. Acad. Sci. U. S. A..

[cit161] Parvini C., Massey M., Mezher M., Cartagena-Rivera A. (2025). Multiplexed Nanoscale Viscoelastic Mapping at Multiple Time Scales of Melanoma Cells as a Label-Free Cancer Biomarker. ACS Nano.

[cit162] Fortes Brollo M. E., Domínguez-Bajo A., Tabero A., Domínguez-Arca V., Gisbert V., Prieto G., Johansson C., Garcia R., Villanueva A., Serrano M. C., Morales M. d. P. (2020). Combined Magnetoliposome Formation and Drug Loading in One Step for Efficient Alternating Current-Magnetic Field Remote-Controlled Drug Release. ACS Appl. Mater. Interfaces.

[cit163] Peng X., Chen K., Liu W., Cao X., Wang M., Tao J., Tian Y., Bao L., Lu G., Teng Z. (2020). Soft Mesoporous Organosilica Nanoplatforms Improve Blood Circulation, Tumor Accumulation/Penetration, and Photodynamic Efficacy. Nano-Micro Lett..

[cit164] Zou D., Wu Z., Yi X., Hui Y., Yang G., Tengjisi Y. L., Wang H., Brooks A., Wang H., Liu X., Xu Z. P., Roberts M. S., Gao H., Zhao C. (2023). Nanoparticle elasticity regulates the formation of cell membrane-coated nanoparticles and their nano-bio interactions. Proc. Natl. Acad. Sci. U. S. A..

[cit165] Yang Z., Zhao Y., Zhang X., Huang L., Wang K., Sun J., Chen N., Yin W., Chen S., Zhi H., Xue L., An L., Li R., Dong H., Xu J., Li Y., Li Y. (2024). Nano-mechanical Immunoengineering: Nanoparticle Elasticity Reprograms Tumor-Associated Macrophages *via* Piezo1. ACS Nano.

[cit166] Xia D., Zhang S., Nielsen E., Ivarsen A. R., Liang C., Li Q., Thomsen K., Ø Hjortdal J., Dong M. (2016). The Ultrastructures and Mechanical Properties of the Descement's Membrane in Fuchs Endothelial Corneal Dystrophy. Sci. Rep..

[cit167] Miranda A., Gómez-Varela A. I., Stylianou A., Hirvonen L. M., Sánchez H., De Beule P. A. A. (2021). How did correlative atomic force microscopy and super-resolution microscopy evolve in the quest for unravelling enigmas in biology?. Nanoscale.

[cit168] Schächtele M., Kemmler J., Rheinlaender J., Schäffer T. E. (2022). Combined High-Speed Atomic Force and Optical Microscopy Shows That Viscoelastic Properties of Melanoma Cancer Cells Change during the Cell Cycle. Adv. Mater. Technol..

[cit169] Ganser C., Nishiguchi S., Chan F., Uchihashi T. (2025). A look beyond topography: Transient phenomena of Escherichia coli cell division captured with high-speed in-line force mapping. Sci. Adv..

[cit170] Galluzzi M., Zhang B., Zhang H., Wang L., Lin Y., Yu X., Chu Z., Li J. (2021). Unveiling a Hidden Event in Fluorescence Correlative Microscopy by AFM Nanomechanical Analysis. Front. Mol. Biosci..

[cit171] Wang L., Wang H., Wagner M., Yan Y., Jakob D. S., Xu X. G. (2017). Nanoscale simultaneous chemical and mechanical imaging *via* peak force infrared microscopy. Sci. Adv..

[cit172] Kolmogorov V. S., Erofeev A. S., Woodcock E., Efremov Y. M., Iakovlev A. P., Savin N. A., Alova A. V., Lavrushkina S. V., Kireev I. I., Prelovskaya A. O., Sviderskaya E. V., Scaini D., Klyachko N. L., Timashev P. S., Takahashi Y., Salikhov S. V., Parkhomenko Y. N., Majouga A. G., Edwards C. R. W., Novak P., Korchev Y. E., Gorelkin P. V. (2021). Mapping mechanical properties of living cells at nanoscale using intrinsic nanopipette–sample force interactions. Nanoscale.

[cit173] Swierczewski M., Chenneviere A., Lee L., Maroni P., Bürgi T. (2023). Nanomechanical and structural study of Au38 nanocluster Langmuir-Blodgett films using bimodal atomic force microscopy and X-ray reflectivity. J. Colloid Interface Sci..

[cit174] Al-Rekabi Z., Contera S. (2018). Multifrequency AFM Reveals Lipid Membrane Mechanical Properties and the Effect of Cholesterol in Modulating Viscoelasticity. Proc. Natl. Acad. Sci. U. S. A..

[cit175] Uluutku B., López-Guerra E. A., Solares S. D. (2021). A new method for obtaining model-free viscoelastic material properties from atomic force microscopy experiments using discrete integral transform techniques. Beilstein J. Nanotechnol..

[cit176] Nguyen H. K., Shundo A., Liang X., Yamamoto S., Tanaka K., Nakajima K. (2022). Unraveling Nanoscale Elastic and Adhesive Properties at the Nanoparticle/Epoxy Interface Using Bimodal Atomic Force Microscopy. ACS Appl. Mater. Interfaces.

[cit177] McCraw M. R., Uluutku B., Solomon H. D., Anderson M. S., Sarkar K., Solares S. D. (2023). Optimizing the accuracy of viscoelastic characterization with AFM force-distance experiments in the time and frequency domains. Soft Matter.

[cit178] Adam C. E., Piacenti A. R., Waters S. L., Contera S. (2024). Enhancing nanoscale viscoelasticity characterization in bimodal atomic force microscopy. Soft Matter.

[cit179] Fricke L. V., Wansink N., Rosso M., Staufer U., Belardinelli P., Alijani F. (2025). Probing Viscoelasticity of Polymeric Coatings Using Nonlinear Dynamic Atomic Force Microscopy. Small Methods.

[cit180] Shim J., Kim C., Lee M., An S., Jhe W. (2024). Multiscale rheology from bulk to nano using a quartz tuning fork-atomic force microscope. Rev. Sci. Instrum..

[cit181] Yang L., Nickmilder P., Verhoogt H., Hoeks T., Leclère P. (2024). Probing Viscoelastic Properties and Interfaces in High-Density Polyethylene Vitrimers at the Nanoscale Using Dynamic Mode Atomic Force. ACS Appl. Mater. Interfaces.

[cit182] Guerrero C. R., Garcia P. D., Garcia R. (2019). Subsurface Imaging of Cell Organelles by Force Microscopy. ACS Nano.

[cit183] Roduit C., Sekatski S., Dietler G., Catsicas S., Lafont F., Kasas S. (2009). Stiffness Tomography by Atomic Force Microscopy. Biophys. J..

[cit184] Stühn L., Fritschen A., Choy J., Dehnert M., Dietz C. (2019). Nanomechanical sub-surface mapping of living biological cells by force microscopy. Nanoscale.

[cit185] Efremov Y. M., Suter D. M., Timashev P. S., Raman A. (2022). 3D nanomechanical mapping of subcellular and sub-nuclear structures of living cells by multi-harmonic AFM with long-tip microcantilevers. Sci. Rep..

[cit186] Walter K., Bourquin J., Amiri A., Scheer N., Dehnert M., Eichhorn A. L., Dietz C. (2023). Probing local lateral forces of focal adhesions and cell-cell junctions of living cells by torsional force spectroscopy. Soft Matter.

[cit187] Magerle R., Dehnert M., Voigt D., Bernstein A. (2020). Nanomechanical 3D Depth Profiling of Collagen Fibrils in Native Tendon. Anal. Chem..

[cit188] Hoffer M., Petersein F., Dehnert M., Lintner T. A., Dietz C. (2025). Anisotropic Subsurface Inter- and Intramolecular Properties of Heterogeneous Polymers Revealed by Torsional Force Spectroscopy. Langmuir.

[cit189] Vilhena J. G., Ortega M., Uhlig M. R., Garcia R., Pérez R. (2021). Practical Guide to Single-Protein AFM Nanomechanical Spectroscopy Mapping: Insights and Pitfalls As Unraveled by All-Atom MD Simulations on Immunoglobulin G. ACS Sens..

[cit190] Morozov I. A. (2021). Atomic force microscopy nanoindentation kinetics and subsurface visualization of soft inhomogeneous polymer. Microsc. Res. Tech..

[cit191] Argatov I. I., Sabina F. J. (2023). Indentation stiffness tomography of fibrous inhomogeneities — An asymptotic model. Int. J. Eng. Sci..

[cit192] Penedo M., Miyazawa K., Okano N., Furusho H., Ichikawa T., Alam M. S., Miyata K., Nakamura C., Fukuma T. (2021). Visualizing intracellular nanostructures of living cells by nanoendoscopy-AFM. Sci. Adv..

[cit193] Ichikawa T. , et al., Probing nanomechanics by direct indentation using Nanoendoscopy-AFM reveals the nuclear elasticity transition in cancer cells, 10.1101/2024.12.13.626302 PMC12560078 41164208

[cit194] Sharahi H. J., Janmaleki M., Tetard L., Kim S., Sadeghian H., Verbiest G. J. (2021). Acoustic subsurface-atomic force microscopy: Three-dimensional imaging at the nanoscale. J. Appl. Phys..

[cit195] Imada H., Kimura K., Onishi H. (2013). Water and 2-Propanol Structured on Calcite (104) Probed by Frequency-Modulation Atomic Force Microscopy. Langmuir.

[cit196] Zhou S., Panse K. S., Motevaselian M. H., Aluru N. R., Zhang Y. (2020). Three-Dimensional Molecular Mapping of Ionic Liquids at Electrified Interfaces. ACS Nano.

[cit197] Seibert S., Klassen S., Latus A., Bechstein R., Kühnle A. (2020). Origin of Ubiquitous Stripes at the Graphite–Water Interface. Langmuir.

[cit198] Nakouzi E., Stack A. G., Kerisit S., Legg B. A., Mundy C. J., Schenter G. K., Chun J., De Yoreo J. J. (2021). Moving beyond the Solvent-Tip Approximation to Determine Site-Specific Variations of Interfacial Water
Structure through 3D Force Microscopy. J. Phys. Chem. C.

[cit199] Yurtsever A., Yoshida T., Badami Behjat A., Araki Y., Hanayama R., Fukuma T. (2021). Structural and mechanical characteristics of exosomes from osteosarcoma cells explored by 3D-atomic force microscopy. Nanoscale.

[cit200] Arvelo D. M., Uhlig M. R., Comer J., García R. (2022). Interfacial layering of hydrocarbons on pristine graphite surfaces immersed in water. Nanoscale.

[cit201] Auer A., Eder B., Giessibl F. J. (2023). Electrochemical AFM/STM with a qPlus sensor: A versatile tool to study solid-liquid interfaces. J. Chem. Phys..

[cit202] Nakouzi E., Kerisit S., Legg B. A., Yadav S., Li D., Stack A. G., Mundy C. J., Chun J., Schenter G. K., De Yoreo J. J. (2023). Solution Structure and Hydration Forces between Mica and Hydrophilic *Versus* Hydrophobic Surfaces. J. Phys. Chem. C.

[cit203] Bao Y., Nishiwaki Y., Kawano T., Utsunomiya T., Sugimura H., Ichii T. (2024). Molecular-Resolution Imaging of Ionic Liquid/Alkali Halide Interfaces with Varied Surface Charge Densities *via* Atomic Force Microscopy. ACS Nano.

[cit204] Arvelo D. M., Comer J., Schmit J., Garcia R. (2024). Interfacial Water Is Separated from a Hydrophobic Silica Surface by a Gap of 1.2 nm. ACS Nano.

[cit205] Arvelo D. M., Garcia-Sacristán C., Chacon E., Tarazona P., Garcia R. (2024). Interfacial water on collagen nanoribbons by 3D AFM. J. Chem. Phys..

[cit206] Li H., Xu Z., Li J., Siria A., Ma M. (2025). Evolution of Interfacial Hydration Structure Induced by Ion Condensation and Correlation Effects. Angew. Chem., Int. Ed..

[cit207] Reischl B., Watkins M., Foster A. S. (2013). Free Energy Approaches for Modeling Atomic Force Microscopy in Liquids. J. Chem. Theory Comput..

[cit208] Miyazawa K., Kobayashi N., Watkins M., Shluger A. L., Amano K., Fukuma T. (2016). A relationship between three-dimensional surface hydration structures and force distribution measured by atomic force microscopy. Nanoscale.

[cit209] Hashimoto K., Amano K., Nishi N., Onishi H., Sakka T. (2021). Comparison of atomic force microscopy force curve and solvation structure studied by integral equation theory. J. Chem. Phys..

[cit210] Benaglia S., Uhlig M. R., Hernández-Muñoz J., Chacón E., Tarazona P., Garcia R. (2021). Tip Charge Dependence of Three-Dimensional AFM Mapping of Concentrated Ionic Solutions. Phys. Rev. Lett..

